# Optimization of Conventional and Ultrasound-Assisted Extraction to Maximize Recovery of Total Phenolic Content and In Vitro Antioxidant Activity from *Crataegus almaatensis* Leaves

**DOI:** 10.3390/antiox14081003

**Published:** 2025-08-16

**Authors:** Zhanar Nabiyeva, Akerke Kulaipbekova, Serena Carpentieri, Yuliya Pronina, Abdyssemat Samadun, Elmira Assembayeva, Giovanna Ferrari

**Affiliations:** 1 Research Institute of Food Safety, Almaty Technological University, Almaty 050012, Kazakhstan; atu_nabiyeva@mail.ru (Z.N.); abdu.93_93@mail.ru (A.S.); 2 Department of Food Technology, Almaty Technological University, Almaty 050012, Kazakhstan; medvezhonok_87@inbox.ru (Y.P.); elmiraasembaeva@mail.ru (E.A.); 3 Department of Industrial Engineering, University of Salerno, 84084 Fisciano, Italy; gferrari@unisa.it; 4 ProdAl Scarl, University of Salerno, 84084 Fisciano, Italy

**Keywords:** hawthorn leaves, extraction, ultrasound, bioactive compounds, optimization, HPLC-DAD, LC-Q/TOF-MS

## Abstract

Background: *Crataegus almaatensis*, an endemic hawthorn species from Kazakhstan, is known for its rich content of phenolic compounds and flavonoids with significant pharmacological potential. This study aimed to optimize and compare conventional solid–liquid extraction (SLE) and ultrasound-assisted extraction (UAE) processes for maximizing the extractability of bioactive compounds from hawthorn leaves powder. Methods: The effects of temperature, extraction time, ethanol concentration, and solid-to-liquid ratio (or ultrasound power in the case of UAE) on total phenolic content (TPC), total flavonoid content (TFC), and antioxidant activity (FRAP, DPPH, and ABTS assays) were systematically evaluated. Results: The UAE method yielded higher concentrations of TPC and TFC, with up to 16% improvement in TPC and reduced ethanol usage (40% (*v*/*v*)) compared to SLE (75% (*v*/*v*)), demonstrating its efficiency and sustainability. Optimal extraction conditions were identified as 70 °C, 75% ethanol, 34 min, and an S/L ratio of 0.05 g/mL for SLE, 70 °C, 40% ethanol, 44 min, and 100 W US power for UAE. High-resolution HPLC-DAD and LC-Q/TOF-MS analyses confirmed the presence of key phenolic acids and flavonoid glycosides, including chlorogenic acid and apigenin-8-C-glucoside-2′-rhamnoside as the most abundant compounds identified. Conclusions: These findings validate UAE as an innovative, eco-friendly method for extracting bioactive compounds from hawthorn leaves and highlight its potential for developing natural antioxidants for pharmaceutical and nutraceutical applications.

## 1. Introduction

Hawthorn (*Crataegus* spp.) is a flowering plant in the rose family (Rosaceae), which includes various species native to temperate latitudes of the Northern Hemisphere, including Europe, Asia, and North America.

The main morphological characteristics of hawthorn are its shrubby or tree-like form, resistance to various climatic conditions, and ability to grow on a wide range of soils, including stony and rubbly substrates [1]. In South Kazakhstan, particularly in the Zailiyskiy Alatau, *Crataegus altaica*, *Crataegus almaatensis*, and *Crataegus ambigua* species are found growing in mountain areas, gorges, and river valleys, preferring well-drained soils [2]. Hawthorn berries are the main edible part of hawthorn, traditionally used in foods (juice, jams, teas, supplements). The leaves are byproducts derived from hawthorn juice and jam production processes, often harvested alongside the berries and subsequently used in herbal infusions and teas, extracts for supplements (cardiovascular support), flavorings, or functional ingredients. Biologically active compounds, such as flavonoids (e.g., hyperoside, vitexin), oligomeric procyanidins, and phenolic acids, contained in the fruits, leaves, and flowers of hawthorn have antioxidant properties, contributing to the strengthening of blood vessels, improving blood circulation, and maintaining cardiovascular system health. *Crataegus almaatensis* has been previously characterized by determining its chemical composition, biological activity, and adaptive properties. Several studies [3,4,5] confirmed the presence of many biologically active components in *Crataegus almaatensis* leaves, which can be used for the prevention and treatment of various diseases, including liver diseases. Phytochemical studies conducted by Soares et al. (2019) [2] demonstrated that *Crataegus almaatensis* leaf extracts contain key flavonoids, including hyperoside, quercitrin, and afzelin. Pharmacological studies showed that the aqueous extract (APCa) had vasodilatory activity, whereas the ethanolic extract (CECa) promoted an increase in vascular tone. In addition, APCa showed marked anti-inflammatory properties. Studies conducted by Bekbolatova et al. (2018) [6] provided a detailed analysis of the phenolic composition and antioxidant potential of different parts of *Crataegus almaatensis*. Compared with the European species *Crataegus oxyacantha* L., *Crataegus almaatensis* revealed the presence of 22 phenolic compounds, including flavonoids and phenolic acids. Among them, hyperoside is one of the major flavonoids, while chlorogenic acid is the main phenolic compound in leaves and flowers. Moreover, leaf extracts showed higher antioxidant activity compared to that detected in the fruit extracts. Therefore, this species can be considered to be a promising source for the extraction and isolation of bioactive substances for potential pharmacological and food applications. Conventional extraction processes of bioactive compounds from plant materials face significant limitations due to the presence of plant cell walls and membranes, which act as a physical barrier, impeding the efficient recovery of targeted intracellular compounds and often requiring longer extraction times, higher solvent volumes, and temperatures. This challenge has prompted researchers to investigate the application of various cell disruption techniques aimed at weakening or breaking the plant cell envelope. Ultrasound-assisted extraction (UAE) [7,8], extraction with supercritical CO_2_ [9] microwave-assisted extraction [10] were recently investigated to recover antioxidant compounds from hawthorn leaves and fruits. Such approaches facilitate the release of intracellular compounds, thereby improving extraction efficiency while reducing solvent usage and processing time. Among the cell disruption technologies, ultrasound (US) has emerged as a promising technology for enhancing the recovery of intracellular compounds from plant matrices. The application of ultrasonic waves generates cavitation effects that disrupt plant cell walls, thereby accelerating the release of valuable phytochemicals into the extraction medium. UAE has demonstrated significant potential in enhancing the recovery of phenolic compounds from hawthorn leaves [11,12,13,14], offering a green and efficient alternative to traditional extraction methods. However, the effective application of UAE requires a well-defined optimization process to fully realize its advantages over conventional solid–liquid extraction (SLE) processes. Parameters such as US power, extraction time, solvent-to-solid ratio, temperature, and solvent composition must be finely tuned to achieve maximum extractability and quality of the extracted compounds. Despite the growing interest in hawthorn leaves, a limited number of studies focused on the optimization of UAE parameters to recover bioactive compounds from different species of Crataegus leaves, such as *Crataegus pinnatifida* [11,12] and *Crataegus monogyna* [13]. Moreover, not all relevant factors were optimized comprehensively, and a direct comparison between UAE and conventional extraction methods, which would be essential to assess the benefits of using US technology, was not reported [11,13,14].

The aim of this study was to optimize SLE and UAE processing conditions, investigating the effect of temperature, extraction time, ethanol concentration, and solid-to-liquid ratio (or ultrasound power in the case of UAE) on the phenolic content, flavonoid content, and antioxidant activity of the extracts from *Crataegus almaatensis* leaves. The phenolic composition of the obtained extracts was assessed using HPLC-DAD and LC-Q/TOF-MS analyses.

## 2. Materials and Methods

### 2.1. Raw Materials and Chemicals

As part of the IRN AP23489874 project, “Development of gentle technology for production of natural extracts on processing of local plant raw materials for enrichment of sweets” from 5 August 2024, to 24 August 2024, aerial parts of the hawthorn (*Crataegus almaatensis*)—including leaves, stems, and berries—were collected from the mountainous area of Ile-Alatau National Park. To determine the optimal extraction conditions for bioactive compounds, experiments were conducted on ultrasonic extraction from hawthorn (*Crataegus almaatensis*) leaves. Before extraction, hawthorn leaves were pre-sorted, debris was removed, and damaged or diseased leaves were discarded. The leaves were then dried using the shade-drying method in well-ventilated rooms on silk screens with a layer thickness of 2–3 cm, away from direct sunlight, at a temperature of 25–30 °C for 12–15 days until the moisture content reached approximately 8% (AACC 44-15.02 method). During the first two days, the leaves were turned over 1–2 times daily; thereafter, they were turned every other day to ensure uniform drying.

Once dried, the leaves were sorted and ground using a GM 200 knife mill (RETSCH, Haan, Germany) to a particle size of 0.5–1 mm to increase the contact surface area with the extractant. The crushed raw material was then packed into clean, airtight glass containers and stored in well-ventilated rooms at temperatures between 20 and 25 °C, away from sunlight. Under these storage conditions, the shelf life of the crushed raw material without loss of quality is up to 7 months.

Ethanol ≥ 99.8%, methanol ≥ 99.9%, Folin–Ciocalteu, sodium carbonate ≥ 99.5%, aluminum chloride hexahydrate 99%, sodium hydroxide ≥ 97%, TPTZ (2,4,6-Tris(2-pyridyl)-s-triazine) ≥ 98%, sodium acetate ≥ 99%, acetic acid ≥ 99.99%, iron(III) chloride hexahydrate ≥ 98%, DPPH (1,1-diphenyl-2-picrylhydrazyl) ≥ 95%, gallic acid ≥ 98%, quercetin ≥ 99%, ascorbic acid ≥ 99%, chlorogenic acid ≥ 95%, apigenin-8-C-glucoside-2′-rhamnoside ≥ 99%, epicatechin ≥ 99%, and catechin ≥ 99% were purchased from Sigma Aldrich (Steinheim, Germany).

### 2.2. Ultrasound (US) Equipment

US treatments of hawthorn leaves powder were conducted using a US processor UP 400S (Hielscher GmbH, Chamerau, Germany) with a maximum power of 400 W, a constant frequency of 24 kHz, a Tip H3 (titanium, 3 mm in diameter) probe, and an acoustic power density of 460 W cm^−2^.

### 2.3. Solid–Liquid Extraction (SLE)

The conventional SLE process was carried out using simple maceration on a magnetic stirring and heating plate (IKA^®^ C-MAG HS 7, IKA-Werke GmbH & Co. KG, Staufen im Breisgau, Germany) set to 300 rpm, under the processing conditions described in the following Section 2.4.

### 2.4. Experimental Design

Response surface methodology was used to determine the optimal conditions of the conventional solid–liquid extraction process (SLE) and ultrasound-assisted extraction (UAE), which maximize the extractability of total phenolic compounds, flavonoids, and antioxidant activity (FRAP and DPPH) of hawthorn leaves powder extracts.

A four-factor face-centered central composite design (FC-CCD) was employed to optimize both solid–liquid extraction (SLE) and ultrasound-assisted extraction (UAE) processes. For the SLE process, the following parameters and ranges were selected based on preliminary experiments: extraction temperature (X_1_, 25–70 °C), extraction time (X_2_, 10–90 min), ethanol concentration (X_3_, 0–80%, *v*/*v*) in a water–ethanol mixture, and solid-to-liquid ratio (X_4_, 0.05–0.1 g/mL). The response variables were total phenolic content (Y_1_), flavonoid content (Y_2_), and antioxidant activity assessed by FRAP (Y_3_), DPPH (Y_4_), and ABTS (Y_5_) assays. The experimental design used is shown in Table 1.

Based on the optimized SLE conditions, the same FC-CCD structure (25 runs; Table 2) was applied to the UAE process with adjusted factors appropriate for ultrasonic treatment: input power (X_1_, 100–400 W), extraction temperature (X_2_, 40–70 °C), extraction time (X_3_, 30–90 min), and ethanol concentration (X_4_, 40–70%, *v*/*v*). The solid-to-liquid ratio was fixed at 0.05 g/mL to reduce variability from this factor during ultrasonic treatment. The same response variables (Y_1_–Y_5_) were measured for comparison. During the extraction process, the flask containing the sample was placed in a thermostatic water bath to maintain a constant temperature. The sample temperature was monitored at regular intervals throughout the extraction process using a digital thermometer.

For both SLE and UAE processes, a second-order polynomial model, reported in Equation (1), was reduced by stepwise elimination and utilized to fit the experimental data. Specifically, terms with *p*-values greater than 0.05 were systematically excluded using a stepwise backward elimination approach, as implemented by the Design Expert version 12 software.

(1) $$Y_{k}=\alpha_{0}+{\sum_{i=1}^{5}\alpha_{i}X}_{i}+\sum_{i=1}^{5}\alpha_{ii}X_{i}^{2}+\sum_{i=1}^{5}\sum_{j=i+1}^{5}\alpha_{ij}X_{i}X_{j}$$

where *Y_k_* is the predicted response variable; *X_i_* and *X_j_* are the independent variables; α_0_, *α_i_*, *α_ii_*, and *α_ij_* are the intercept, regression coefficients of the linear, quadratic, and interaction terms of the model, respectively. The number of candidate terms initially considered was 14 for both SLE and UAE, and after the elimination of nonsignificant terms, 5, 2, 6, 3, 5 terms were retained in the final reduced model for TPC, TFC, FRAP, DPPH, ABTS, in the case of SLE, and 6, 3, 2, 4, 2 terms were retained in the final reduced model for TPC, TFC, FRAP, DPPH, ABTS, in the case of UAE.

To determine the optimal extraction conditions, a multi-response optimization was conducted using the desirability function available in Design Expert software. The goal was to maximize all the responses and identify the most favorable combination of extraction parameters.

### 2.5. Analysis of the Extracts

#### 2.5.1. Extraction Yield

At the end of the extraction processes, the extracts were centrifuged at 5700× *g* for 20 min (PK121R model, ALC International, Cologno Monzese, Milan, Italy) to obtain clear supernatants. The supernatants obtained were further concentrated in a R-200/205 Rotavapor (BÜCHI Labortechnik AG, Flawil, Switzerland) until a decrease in volume of up to 90% was achieved. The concentrated extracts were freeze-dried using a 25 L VirTis Genesis freeze-drier (SP Scientific, Gardiner, NY, USA). The dry extract was packed under vacuum in plastic–aluminum pouches and stored under refrigerated conditions (8 °C).

#### 2.5.2. Total Phenolic Content (TPC)

The Folin–Ciocalteau assay was used to evaluate the TPC of hawthorn leaves powder extracts as reported by Carpentieri et al. (2022) [15]. A 1 mL portion of the diluted supernatant was combined with 5 mL of 10% (*v*/*v*) Folin–Ciocalteu reagent and left at room temperature for 5 min. Then, 4 mL of 7.5% (*w*/*v*) sodium carbonate was added. After mixing, the solution was incubated in the dark at room temperature for one hour. The absorbance was measured at 765 nm using a V-650 spectrophotometer (Jasco Inc., Easton, MD, USA). Calibration curves were prepared using gallic acid standards (1–100 mg/L). The TPC was expressed as milligrams of gallic acid equivalents (GAE) per g of dry weight (g_DM_) of hawthorn leaves powder.

#### 2.5.3. Total Flavonoid Content (TFC)

Aluminum chloride assay was used to determine the TFC of hawthorn leaves powder extracts as previously reported by Carpentieri et al. (2022) [15]. A total of 1 mL of the supernatant was combined with 4 mL of distilled water, followed by the addition of 0.3 mL of 5% (*w*/*v*) sodium nitrite. The mixture was kept in the dark for 5 min, after which 0.3 mL of 10% (*w*/*v*) aluminum chloride hexahydrate (AlCl_3_·6H_2_O) in water was added. After 5 min of incubation, 2 mL of 1.0 M sodium hydroxide was introduced, and the final volume was brought up to 10 mL with distilled water. Standard quercetin solutions (20–100 μg/mL) were prepared using the same procedure. Absorbance of both samples and standards was measured at 510 nm. The TFC was expressed as mg of quercetin equivalent (QE) per g_DM_ of hawthorn leaves powder.

#### 2.5.4. Ferric Reducing Antioxidant Power (FRAP)

The FRAP assay for hawthorn leaves powder extracts was performed following the method described by Benzie and Strain (1996) [16]. In brief, 0.5 mL of the diluted supernatant was mixed with 2.5 mL of FRAP reagent and incubated for 10 min. The absorbance was then recorded at 593 nm. Ascorbic acid, in concentrations ranging from 0 to 2 mmol/L, was used to construct the standard calibration curve. The FRAP values were expressed as mg of ascorbic acid equivalents (mg AAE) per g_DM_ of hawthorn leaves powder.

#### 2.5.5. Free-Radical-Scavenging Capacity (DPPH)

The DPPH assay was used to evaluate the free-radical-scavenging capacity of the extracts [17]. Briefly, 3.9 mL of a 25 ppm DPPH solution in methanol was mixed with 0.1 mL of supernatant and incubated in the dark for 5 min. The absorbance of the mixture was read at a wavelength of 515 nm. The inactivation level of extracts was evaluated as reported in the following equation (Equation (2)):

(2) $$\%I=100-\left(\frac{{ABS}_{S}}{{ABS}_{B}}\right)*100\%$$

where *ABS_s_* is the absorbance of the sample and *ABS_B_* is the absorbance of the blank.

The antioxidant activity was expressed as both percentage of DPPH radical inhibition (%I_DPPH_) and IC_50_ values, which were estimated by linear interpolation between two concentrations bracketing 50% inhibition, using data across a concentration range of the extract of 0.05 to 1.00 mg/mL.

#### 2.5.6. ABTS Scavenging Activity

The ABTS (2,2-azo-bis (3-ethyl benzothiazoline-6-sulfonic acid)) assay was used to evaluate the scavenging activity of extracts following the methodology of Olędzki et al. (2022) [18]. The absorbance of the reacting mixture was recorded at 734 nm. Ascorbic acid was used as a standard to generate the calibration curve (100–800 μmol/L). The antiradical capacity was expressed as mg of ascorbic acid equivalents (mg AAE) per g_DM_ of hawthorn leaves powder.

#### 2.5.7. HPLC-DAD Analysis

The High-Performance Liquid Chromatography—Diode Array Detection (HPLC-DAD) analyses of the hawthorn leaves powder extracts obtained at the optimal extraction conditions were performed as reported by Rrucaj et al. (2024) [19]. The separation of the extracts was carried out using a Vanquish HPLC System (Thermo Fisher Scientific, Waltham, MA, USA) equipped with a Luna C18 reverse phase column, 250 mm × 4.6 mm with a 5 μm particle size (Phenomenex, Torrance, CA, USA), at 37 °C. The following gradient of the solvent B (ACN/0.1% TFA) was used to carry out the separations at a 1.0 mL min^−1^ constant flow rate: 0–4 min: 0% B; 4–14 min 0–14% B; 14–30 min 14–28% B; 30–34 min 28% B; 34–42 min 28–60% B; 42–45 min 60–80% B; 45–50 min 80–100% B. Solvent A consisted of 0.1% TFA in HPLC grade water. The injection volume of the extract was 50 μL. A diode array detector (DAD) was used to record the UV-vis spectra every 2 s in the 190–650 nm range. The HPLC separations were monitored by recording the λ = 520, 360, 320, and 280 nm wavelengths. Data were processed using the Chromeleon™ Chromatography Data System (CDS) Software, version 7 (Thermo Fisher Scientific). Standard solutions, in the range of 5–250 mg kg^−1^ in methanol and ten-fold diluted with 0.1% TFA in water prior to injection, were used to build calibration curves to quantify the phenolic compounds (R^2^ > 0.99).

#### 2.5.8. LC-Q/TOF-MS Analysis

The chromatographic analysis was carried out using an Agilent Technologies (Santa Clara, CA, USA) 1260 Infinity Series LC coupled with an Agilent Technologies 6530 UHD Accurate Mass LC-Q/TOF-MS, equipped with an electrospray ionization Agilent Technologies Dual Jet Stream ion source (Dual AJS ESI). Chromatographic separation was performed with a 250 mm × 2.1 mm i.d. C18 reversed-phase Aeris core-shell column, 3.6 μm particle diameter (Phenomenex, Torrance, CA, USA), and 50 μL was the volume of injection. The column temperature was maintained at 37 °C during the LC analyses. The solvents were 0.1% formic acid in water, Milli-Q (A), and 0.1% formic acid in acetonitrile (B), at a flow rate of 0.2 mL min^−1^, with the following gradient: 5–60% B in 60 min after 5 min isocratic elution at 5% B. The Q/TOF-MS conditions were the following: drying gas flow (N_2_), 12.0 L min^−1^; nebulizer pressure, 45 psi; gas drying temperature, 350 °C; capillary voltage, 3500 V; fragmentor voltage, 150 V; skimmer voltage 65 V and octopole RF peak, 750 V. Dual AJS ESI interface was used in negative ionization mode and negative ions were acquired in the range of 100–1200 *m*/*z* for MS scans and auto MS/MS scans, at a scan rate of 2 scans s^−1^ for MS and for MS/MS, respectively. Automatic acquisition mode MS/MS was performed using collision energy automatically calculated. Internal mass correction was enabled, using two reference masses at 121.0509 and 922.0098 *m*/*z*. Instrument control and data acquisition were carried out using Agilent MassHunter Workstation software B10.00. All the MS and MS/MS data of the validation standards were integrated by MassHunter Quantitative Analysis B.10.0 (Agilent Technologies). For the putative identification of metabolites in extract, raw MS and MS/MS data were converted into CEF files and processed using the SIRIUS software tool (vers. 6.1.0, Jena University, Germany). Molecular formulas were predicted based on accurate mass and isotope patterns, while MS/MS fragmentation patterns were used for structural elucidation and compound annotation. Identifications were assigned by matching experimental spectra to in silico fragmentation trees and reference compound databases integrated within SIRIUS.

#### 2.5.9. Statistical Analysis

All the analyses of the obtained extracts were performed in triplicate, and the results were reported as means ± standard deviations. Differences among mean values were analyzed by one-way variance (ANOVA) using SPSS 20 (SPSS IBM, Chicago, IL, USA) statistical package. Tukey test was carried out as a post hoc test to determine statistically significant differences (*p* < 0.05). The software package Design Expert Version 12 (Minneapolis, MN, USA) was used to define the experimental design (FC-CCD), as well as to perform the analysis of data including the determination of ANOVA parameters, such as R^2^, adjusted R^2^, predicted R^2^, F-values, lack of fit, *p*-values, adequate precision, sum of squares, degree of freedom, mean square, model term levels of significance, and the determination and validation of the optimal processing conditions using desirability functions. The contribution percentages of each significant term were also calculated by dividing the sum of squares of the individual factor by the total model sum of squares.

## 3. Results and Discussion

### 3.1. Effect of SLE and UAE Processing Conditions on the Extractability of Bioactive Compounds from Hawthorn Leaves Powder

#### 3.1.1. Model Fitting of the Experimental Data (SLE)

Various factors, including type of solvent, extraction temperature, diffusion time, and solid-to-liquid (S/L) ratio, are pivotal in affecting the recovery of valuable intracellular compounds from herbal matrices [20,21,22]. These factors significantly influence the disruption of cellular structures and the solubilization of intracellular constituents, thereby impacting overall extractability. Optimizing these conditions is essential for maximizing the recovery of phenolic compounds, flavonoids, and other antioxidants from herbal matrices.

In this study, the effect of four independent factors, such as extraction temperature, diffusion time, ethanol concentration, and S/L ratio, on TPC, TFC, and antioxidant activity (FRAP, DPPH, and ABTS) of extracts from hawthorn leaves powder achieved from conventional SLE was evaluated to optimize the processing conditions. The values and significance of the regression coefficients of the model and the corresponding *p*-values, adjusted and predicted R^2^, lack of fit, and F-value of the model are reported in Table 3. Equations of reduced models, including significant terms, determination coefficients (R^2^), are reported in Table S1 of the Supplementary Material. The coefficients and *p*-values of the unrefined model have been reported for transparency in Table S3 of the Supplementary Material.

Results show that, for the extracts obtained after SLE of bioactives from hawthorn leaves powder, the extraction temperature resulted in statistically significant linear and quadratic effects on TPC, FRAP, and ABTS values, with a nonsignificant effect detected only for TFC and DPPH.

Although the magnitude of the quadratic term is relatively small, its statistical significance indicates that temperature influences the extraction process in a U-shaped manner. Specifically, the responses initially declined slightly with increasing temperature before rising again at higher temperatures, suggesting a moderate thermal sensitivity of phenolic compounds, balanced by enhanced extractability at elevated temperatures.

This trend is consistent with previous findings across various plant matrices. For instance, Rittisak et al. (2022) [23] observed a mild U-shaped relationship between temperature and antioxidants recovered from roasted rice germ herbal tea, attributing the initial decrease to the thermal degradation of heat-labile phenolics, followed by increased recovery at higher temperatures due to cell wall disruption and improved solubility. Similarly, Dar et al. (2024) [24] identified a significant but low-magnitude positive quadratic term for temperature in the ultrasound-assisted extraction of antioxidants from Nigella sativa seeds.

Flavonoid content was not significantly affected by temperature, implying that flavonoid structures might be more stable under the studied temperature range (25–70 °C) compared to simple phenolic acids.

Ethanol concentration in water was the most significant factor affecting all responses. It showed a positive linear effect and a significant negative quadratic effect on TPC, TFC, FRAP, and ABTS values (*p* < 0.001) of extracts obtained from SLE. This trend indicates an inverted U-shaped relationship, where increasing ethanol percentage initially enhanced extraction efficiency, due to improved solubility and polarity, then, beyond an optimal ethanol concentration, further increases led to a decline in response values.

This type of ethanol-dependent behavior is well-documented in studies investigating the extractability of phenolic compounds from plant-based biomasses, where a mixed-polarity solvent maximized extraction yield [15,25].

The interaction between ethanol percentage and solid-to-liquid ratio showed a notable negative effect (*p* < 0.01) on FRAP values, implying that beyond certain concentrations, increasing both parameters simultaneously reduces the extractability of antioxidant compounds.

Extraction time did not exert significant linear and quadratic effects on the response variables investigated, suggesting that this factor played a secondary role in the recovery of valuable intracellular compounds from hawthorn leaves powder. Indeed, prolonged extraction processes did not enhance the extractability of the responses investigated. In some cases, prolonged extraction can lead to degradation of antioxidants or unnecessary exposure to oxidative conditions [13]. It is worth highlighting that in the case of SLE, the temperature—time interaction coefficient is negative and significant for TFC and DPPH, indicating that increasing both temperature and time does not provide additional benefits to the extraction efficiency.

The S/L ratio exerts highly negative linear effects on TPC, FRAP, and ABTS, indicating a strong inverse relationship between the amount of solid and phenolic compounds and antioxidant activity in the extracts obtained. Interestingly, the S/L ratio had no significant impact on TFC and DPPH values, suggesting that while TPC, FRAP, and ABTS decreased, the flavonoids and specific antioxidant compounds contributing to scavenging activities might not scale proportionally with the amount of plant material in the solvent.

#### 3.1.2. Model Fitting of the Experimental Data (UAE)

The same design was utilized to investigate the effect of extraction temperature, diffusion time, ethanol concentration, and US power on bioactive compounds of extracts from hawthorn leaves powder achieved from the UAE process at a fixed S/L ratio of 0.05 g/mL, selected according to the optimized SLE processing conditions.

The values and significance of the regression coefficients of the model and the corresponding *p*-values, adjusted and predicted R^2^, lack of fit, and F-value of the model are reported in Table 4. Equations of reduced models, including significant terms, determination coefficients (R^2^), are reported in Table S2 of the Supplementary Material. The coefficients and *p*-values of the unrefined model have been reported for transparency in Table S4 of the Supplementary Material.

In the case of the UAE process, temperature had a significant linear positive effect on TPC, TFC, and FRAP (*p* < 0.001, *p* < 0.01, *p* < 0.001, respectively), and a quadratic effect on DPPH (*p* < 0.001) showing that an increase in temperature enhanced phenolic compounds, flavonoids extraction and antioxidant activity.

Ethanol showed a strong positive effect on TPC and TFC (*p* < 0.001), similar to SLE, confirming that ethanol is crucial for phenolic solubilization.

However, it negatively affected FRAP values. Regarding the linear dependence of the responses on ethanol concentration, it is interesting to note that a reduced significance was detected for FRAP, DPPH, and ABTS values upon US application compared to SLE. This implies that, in the investigated variable domain, US treatment decreased the effect of ethanol concentration on the extractability of antioxidants. These findings suggest that while ethanol concentration is a critical factor in the extraction of antioxidants, the application of ultrasound may modify its impact, potentially reducing its significance compared to other extraction parameters.

Moreover, the interaction between ethanol percentage and temperature showed a notable negative effect (*p* < 0.01) for TPC and TFC, implying that increasing both parameters simultaneously reduces the recovery of these compounds.

Extraction time did not exert significant linear and quadratic effects on the response variables investigated. Nonetheless, in contrast to the SLE process, the temperature—time interaction coefficient is positive and significant for TPC and DPPH, suggesting a synergistic effect between temperature and time, which justifies the selection of a moderately longer extraction time under optimal conditions (Table 5).

As regards the US power, no significant linear effect on the responses was observed. It showed a slight positive quadratic effect on TPC; however, the small absolute values of the coefficient suggested that most of the bioactive compounds were efficiently extracted at the lowest US power studied (100 W). Increasing US power, no significant enhancements in the extractability of bioactive compounds were detected. These findings align with previous research on the optimization of UAE parameters for extracting phenolic compounds from *Centella asiatica* [26]. These authors found that ethanol concentration and S/L ratio significantly influenced TPC and TFC, while US power had nonsignificant effects.

In addition, the interaction between ethanol concentration and temperature with US power had a positive effect on DPPH values, indicating that these variables synergistically affect the scavenging capacity of extracts.

#### 3.1.3. Model Validation and Accuracy

The Analysis of Variance (ANOVA) revealed that the relationship between the response variables and extraction parameters had determination coefficients (R^2^) ranging from 0.870 to 0.942 for SLE and from 0.886 to 0.955 for UAE, indicating a strong correlation between the experimental data and the values predicted by the model. For both SLE and UAE, the regression models show strong statistical significance across all responses, as indicated by high model F-values (all *p* < 0.01 or 0.001) and nonsignificant lack of fit, confirming good model adequacy. The models demonstrate good fit and predictive power, predicted R^2^ values are in reasonable agreement with the adjusted R^2^ values, with differences lower than 0.08.

This is further supported by the correlation plots of predicted vs. actual values reported in Figures S1 and S2 of the Supplementary material, where a strong alignment along the diagonal demonstrates the accuracy of the model in estimating experimental data. Normal probability plots of residuals are used to assess whether the residuals (errors) from the models follow a normal distribution, which is one of the assumptions for valid regression analysis. These plots are reported in Figures S3 and S4. For TPC, TFC, FRAP, and ABTS, the residuals exhibit an approximately linear trend along the reference line, indicating that the errors are normally distributed. This suggests that the models for these responses are statistically sound and meet the underlying assumptions of ANOVA and regression analysis. DPPH, in the case of SLE, shows slight deviations from linearity, which may indicate minor non-normality or potential heteroscedasticity.

#### 3.1.4. Optimization of SLE and UAE Processing Conditions

**Figure 1:**
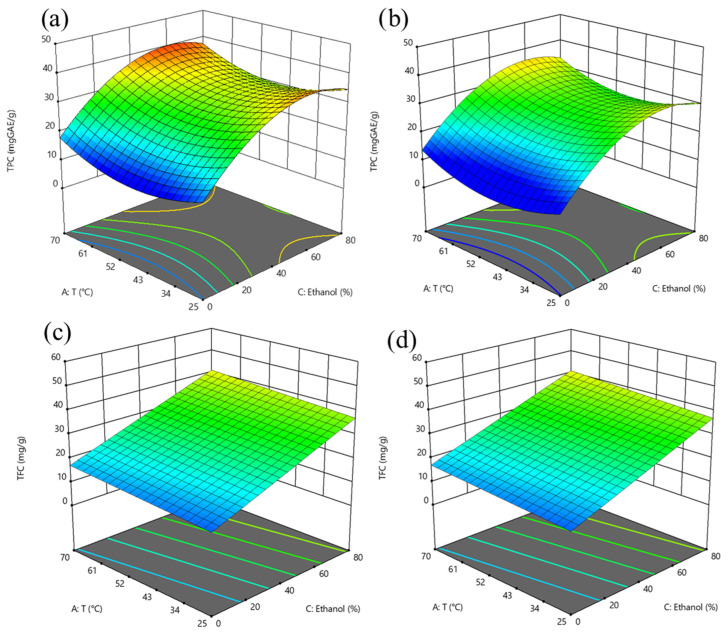
Response surface graphs for TPC (mg GAE/g_DM_) (**a**,**b**) and TFC (mg QE/g_DM_) (**c**,**d**) of extracts from hawthorn leaves powder after SLE as a function of temperature and ethanol concentration, at S/L ratio of 0.05 g/mL (**a**,**c**), and 0.1 g/mL (**b**,**d**). The surface color gradient represents the magnitude of the response: cooler colors (e.g., blue) indicate lower response values, while warmer colors (e.g., yellow to red) correspond to higher response values.

**Figure 2:**
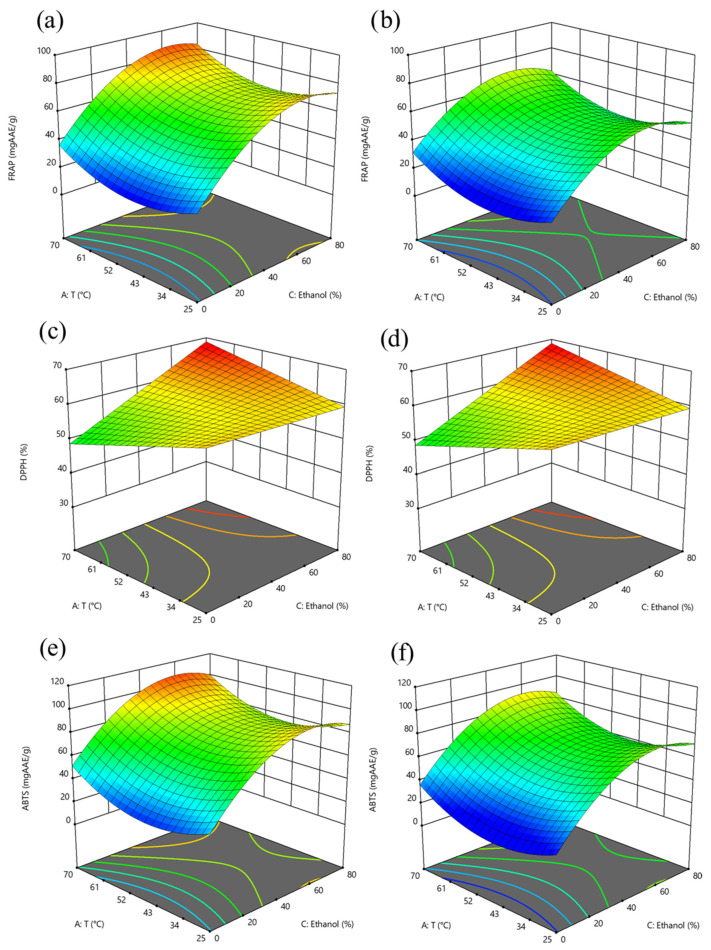
Response surface graphs for FRAP (mg AAE/g_DM_) (**a**,**b**), DPPH (%) (**c**,**d**) and ABTS (mg AAE/g_DM_) (**e**,**f**) of extracts from hawthorn leaves powder after SLE as a function of temperature and ethanol concentration, at S/L ratio of 0.05 g/mL (**a**,**c**,**e**), and 0.1 g/mL (**b**,**d**,**f**). The surface color gradient represents the magnitude of the response: cooler colors (e.g., blue) indicate lower response values, while warmer colors (e.g., yellow to red) correspond to higher response values.

**Figure 3:**
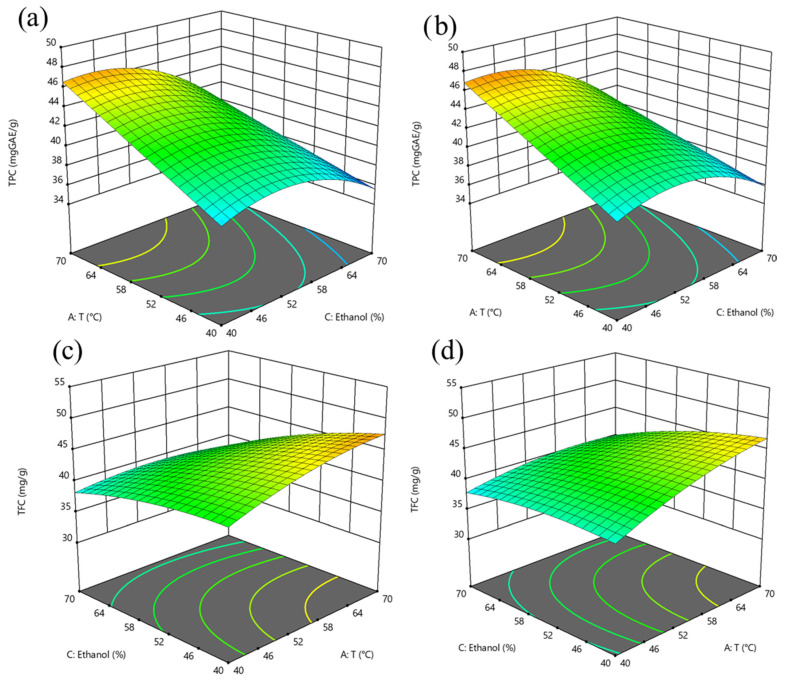
Response surface graphs for TPC (mg GAE/g_DM_) (**a**,**b**) and TFC (mg QE/g_DM_) (**c**,**d**) of extracts from hawthorn leaves powder after UAE as a function of temperature, ethanol concentration, at US power of 100 W (**a**,**c**), and 400 W (**b**,**d**). The surface color gradient represents the magnitude of the response: cooler colors (e.g., blue) indicate lower response values, while warmer colors (e.g., yellow to red) correspond to higher response values.

**Figure 4:**
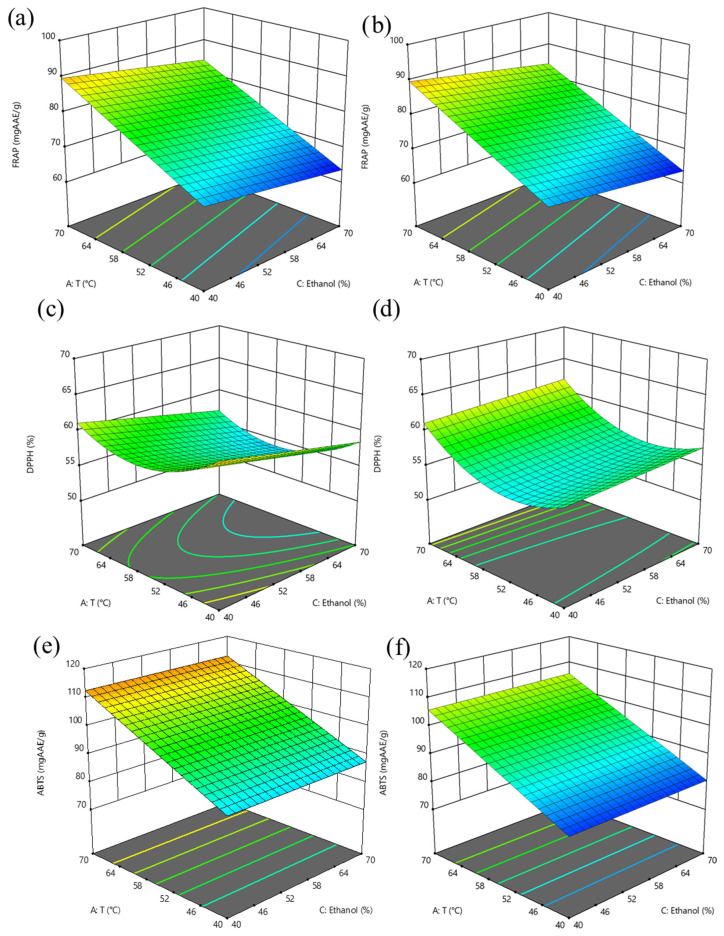
Response surface graphs for FRAP (mg AAE/g_DM_) (**a**,**b**), DPPH (%) (**c**,**d**) and ABTS (mg AAE/g_DM_) (**e**,**f**) of extracts from hawthorn leaves powder after UAE as a function of temperature and ethanol concentration, at US power of 100 W (**a**,**c**,**e**), and 400 W (**b**,**d**,**f**). The surface color gradient represents the magnitude of the response: cooler colors (e.g., blue) indicate lower response values, while warmer colors (e.g., yellow to red) correspond to higher response values.

The 3D response surface graphs showing the interaction between the extraction temperature (25–70 °C), ethanol concentration (0–80%, *v*/*v*) at a S/L ratio of 0.05 g/mL and 0.1 g/mL on TPC, TFC, FRAP, DPPH, and ABTS of the extracts obtained after SLE of hawthorn leaves powder are reported in Figure 1 and Figure 2. Figure 3 and Figure 4 depict the 3D response surface graphs demonstrating the interacting effect between the extraction temperature (25–70 °C), ethanol concentration (40–70%, *v*/*v*) at a US power of 100 W and 400 W, and a S/L ratio of 0.05 g/mL, on TPC, TFC, FRAP, DPPH, and ABTS of the extracts obtained after UAE of hawthorn leaves powder.

Throughout the entire investigated domain, the response variables exhibited a similar trend. The UAE process applied to hawthorn leaves powder resulted in a higher amount of bioactive compounds in the extracts compared to the SLE process. It can be observed that the ethanol concentration in water appeared as the factor that most influenced the investigated responses, as confirmed by the significance of the coefficients (Tables S1 and S2).

Moreover, the influence of ethanol concentration on the extractability of bioactive compounds showed a parabolic trend, which appeared less pronounced when applying UAE compared to the conventional SLE process.

The concentration of ethanol used in the extraction mixture significantly influences the recovery of phenolic compounds from plant materials. Ethanol–water mixtures are widely used for extracting phenolic compounds due to their ability to solubilize a broad spectrum of molecules with varying polarities. By combining both solvents, the resulting mixture offers an intermediate polarity that enhances the overall extraction efficiency of diverse phenolic constituents.

Studies on hawthorn leaves and other medicinal herbs consistently report a parabolic relationship between ethanol concentration and total phenolic content. As ethanol concentration increases, phenolic extraction initially improves due to enhanced solubility of less polar compounds and increased disruption of plant cell membranes. However, beyond a certain ethanol concentration, the concentration begins to decline. This decrease is typically attributed to the reduced ability of high-ethanol mixtures to solubilize more polar phenolics, along with reduced swelling of plant tissues, which hinders solvent penetration [27].

This trend has also been observed in similar herbs and plant materials. For example, in rosemary, optimal phenolic extraction occurred at ethanol concentrations ranging from 30% to 50% (*v*/*v*), depending on the extraction technique used; beyond this range, extraction efficiency decreased [28].

When employing UAE, the optimal ethanol concentration tends to be lower compared to conventional methods. In the case of *Crataegus pinnatifida* (a species of hawthorn) leaves, 39% ethanol (*v*/*v*) has been identified as the optimal concentration for maximizing total phenolic content [11].

Ultrasound enhances extraction by inducing cavitation, which disrupts plant cell walls and facilitates solvent diffusion. This mechanical effect can compensate for the reduced solvating power of lower ethanol concentrations. Likewise, Martin-Garcia et al. (2021) [13], who investigated the optimization of UAE of phenolic compounds from *Crataegus monogyna* leaves, found that the optimal acetone concentration in water (*v*/*v*), ranging from 20% to 80% was 50% (*v*/*v*).

Nevertheless, increasing the US power from 100 W to 400 W did not induce any significant increases in TPC, TFC, FRAP, DPPH, and ABTS values in the extracts (Figure 3 and Figure 4). The quadratic coefficient of US power (Table 4) was positive for TPC with a slight significance and a small absolute value, indicating that extreme power levels are not necessary to enhance the extractability of target phenolic compounds.

Together with the type of solvent, the extraction temperature plays a critical role in the efficiency of phenolic compound recovery from plant matrices. From the 3D response surface graphs, it is possible to observe, for TPC, FRAP, and ABTS (Figure 1 and Figure 2), in the case of SLE, and for DPPH (Figure 4), in the case of UAE, the U-shaped trend, in line with the positive quadratic coefficients of temperature. Higher temperatures can disrupt the structural integrity of plant tissues, significantly enhancing the mass transfer process by increasing the solubility and diffusivity of phenolic compounds in the solvent. As the temperature rises, the surface tension and viscosity of the solvent decrease, allowing it to effectively penetrate plant cell walls and facilitate the release of intracellular bioactive compounds [27]. Several studies have demonstrated that moderate to elevated extraction temperatures improved the extractability of phenolic compounds from various medicinal and aromatic herbs, including hawthorn leaves. For instance, the extraction of phenolics from white tea leaves [29] and rosemary [30,31] showed peak efficiency at extraction temperatures of 65–70 °C. Similarly, Liang et al. (2022) [14] and Pan et al. (2012) [32], who investigated the optimization of UAE of flavonoids from hawthorn leaves and seeds, respectively, found an optimal temperature of 65 °C. However, it is important to note that excessively high temperatures may lead to the breakdown of certain sensitive phenolic compounds, thus lowering the overall antioxidant capacity of the extract, and seeking a compromise between enhanced extractability and compound stability.

Regarding the extraction time, the 3D surface graphs revealed that, regardless of the application of US treatment, this factor scarcely affected TPC, TFC, FRAP, DPPH, and ABTS values in the extracts.

Liang et al. (2022) [14] found a relatively short optimal extraction time of 40 min when investigating the optimization of ultrasonic-assisted aqueous two-phase extraction of flavonoids from hawthorn leaves.

Similarly, other authors observed optimal extraction times ranging from 31 min to 55 min when optimizing the UAE process of phenolic compounds from *Crataegus pinnatifida* and *Crataegus monogyna* leaves [11,13].

Moreover, from the graphs reported in Figure 1 and Figure 2, the lowest S/L ratio investigated (0.05 g/mL) enhanced the extractability of phenolic compounds and flavonoids from hawthorn leaves powder compared to higher S/L ratios. A lower solid content, in the range of 0.03–0.07 g/mL, provides enhanced solvent saturation capacity, while a higher solvent volume increases the contact surface area between the solid and the solvent, reducing viscosity and clumping of powdery leaves and improving solubility and diffusion of solutes [11,14].

Based on the findings obtained and the use of desirability function, the optimal values of the independent variables that maximized the investigated responses were shown by the adopted model to be 70 °C, 75% ethanol–water mixture, 34 min, and S/L ratio 0.05 g/mL for extracts from SLE, and 70 °C, 40% ethanol–water mixture, 44 min, and US power of 100 W for extracts from UAE of hawthorn leaves powder, with desirability values of 0.93 and 0.91, respectively. Under these optimized conditions, the TPC, TFC, FRAP, DPPH, and ABTS predicted and experimental values of the extracts achieved from SLE and UAE processes are reported in Table 5. To validate the model predictions, experimental values were measured under the optimized extraction conditions and compared with the predicted responses. The % deviations between predicted and actual values for TPC, TFC, FRAP, DPPH, and ABTS ranged from 0.52% to 9.84%, supporting the adequacy and predictive capability of the response surface models, indicating a good agreement between experimental and predicted data.

Even though the amount of bioactives extracted from herbal matrices depends on several factors such as variety, growing conditions, harvesting time, post-harvest handling, equipment and experimental protocols, these results appeared consistent with those observed by other authors who found comparable concentrations of phenolic compounds (12.41–82.74 mg GAE/g_DM_) [13,14,33,34], flavonoids (0.97–63.34 mg QE/g_DM_) [33,35], and antioxidant activity (49.62–134.68 mg Trolox/g_DM_) [13] in extracts from different species of hawthorn leaves.

Interestingly, the application of optimized UAE process can be successfully used to intensify the extractability of phenolic compounds (16%), flavonoids (7%), antioxidant activity (FRAP, 7%, DPPH, 4%, and ABTS, 9%) from hawthorn leaves powder with a significantly reduced ethanol concentration compared to conventional SLE process.

This reduction is made possible by the mechanical effects of ultrasound, which enhance cell disruption, mass transfer, and solvent interaction, reducing the dependence on high solvent polarity, making UAE a more sustainable and economical alternative for plant bioactive extraction.

Ultrasound generates high-frequency sound waves that create microbubbles in the solvent. When these bubbles collapse, they produce intense localized energy that disrupts plant cell walls, improves solvent penetration into cellular structures, and effectively releases intracellular phenolic compounds [36].

The results obtained are consistent with those reported by Martin-Garcia et al. [13], who demonstrated that optimized UAE conditions led to a significant enhancement in phenolic compound recovery from *C. monogyna* leaves. Similarly, Luo et al. [11] reported an increase in TPC by approximately 13–18% in *C. pinnatifida* using UAE compared to conventional methods, confirming the high efficiency and versatility of UAE across different *Crataegus* species. The extraction yield, expressed as % dry weight of extract per gram of raw material, was 18% for UAE and 15% for SLE (Solid–Liquid Extraction). This 20% increase in yield using UAE compared to SLE highlights the enhanced efficiency of ultrasound in disrupting plant cell walls and improving mass transfer.

Moreover, the optimal hawthorn leaf extract exhibited an IC_50_ value of 0.35 ± 0.02 mg/mL for SLE and 0.3 ± 0.01 mg/mL for UAE using the DPPH assay, indicating a moderate antioxidant activity. This value is consistent with that found for leaf extracts from commonly used plants for their antioxidant properties, including *Crataegus oxyacantha* [37] and *Crataegus pinnatifida* leaves [11], which have IC_50_ values ranging between 0.04 and 0.69 mg/mL, widely depending on the type of solvent and extraction method used.

The optimized extraction conditions developed in this study offer promising advantages in terms of sustainability and potential scalability for industrial applications. The reduced ethanol concentration not only minimizes solvent consumption and associated environmental impact but also lowers costs related to solvent recovery and handling. Furthermore, UAE operates efficiently at moderate temperatures and extraction times, with relatively low power applied (100 W), contributing to the overall energy efficiency of the process. These features, combined with the ability to enhance extraction yields without the need for harsh conditions or large solvent volumes, support the feasibility of UAE as a more sustainable and economically viable alternative for large-scale production of plant-based bioactives.

#### 3.1.5. Phenolic Composition of the Extracts

HPLC analysis and mass spectroscopy aimed at investigating the phenolic composition of the extracts from hawthorn leaves powder obtained after SLE (70 °C, 75% ethanol–water mixture, 34 min, S/L ratio of 0.05 g/mL) and UAE (70 °C, 40% ethanol–water mixture, 44 min, S/L ratio of 0.05 g/mL, US power of 100 W) under optimized conditions.

**Figure 5:**
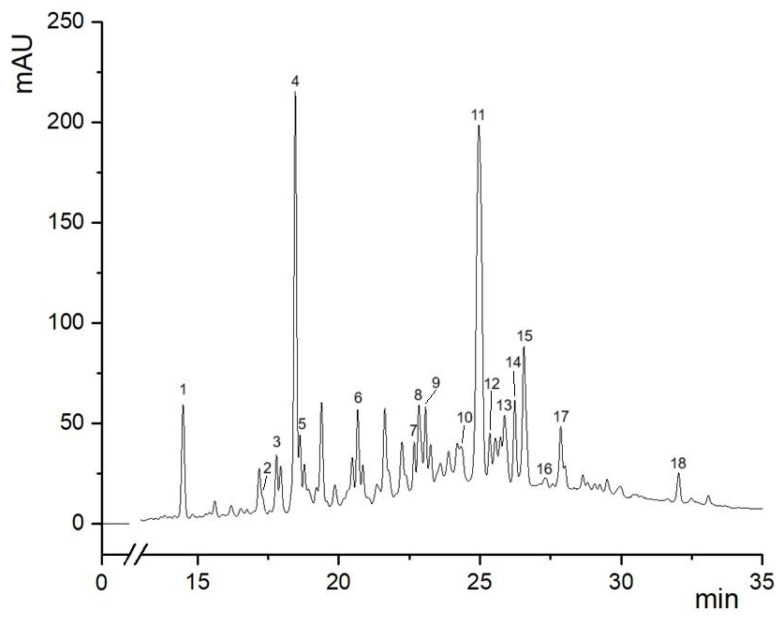
HPLC-DAD profile (280 nm) of the extract obtained after SLE under optimized conditions (temperature of 70 °C, solvent 75% ethanol–water mixture, time of 34 min, and S/L ratio of 0.05 g/mL).

The HPLC-DAD profiles of extracts obtained after SLE and UAE were comparable with concentrations of chlorogenic acid, apigenin-8-C-glucoside-2′-rhamnoside, epicatechin, and catechin reported in Table 6. Notably, the application of ultrasound did not result in any significant alteration or degradation of the HPLC profile compared to the extract obtained via SLE, confirming the structural integrity and preservation of the phenolic composition. The obtained HPLC-DAD profile of the extract is reported in Figure 5, and the identified phenolic compounds are listed in Table 7.

Figure 5 and Table 7 show that the dominant peaks are associated with chlorogenic acid and apigenin-8-*C*-glucoside-2′-rhamnoside, suggesting that these are major bioactive constituents of hawthorn leaves powder extract. Consistently with previous findings [3,38,39], several classes of compounds were putatively identified in hawthorn leaves extracts, such as phenolic acids (neochlorogenic, chlorogenic, cryptochlorogenic acids), flavan-3-ols (catechin, epicatechin, procyanidin C1), flavone/flavonol glycosides (apigenin, luteolin, quercetin derivatives), dihydrochalcones (nothofagin), and lignan (cinchonain 1A).

Chlorogenic acid is a significant phenolic compound found in hawthorn leaves; however, its concentration can vary based on species, extraction methods, and environmental factors. The accumulation of phenolics in leaves, indeed, is commonly suggested to be sensitive to the environment [40]. For instance, a study analyzing methanolic extracts of *Crataegus pinnatifida* reported chlorogenic acid content at approximately 0.84 mg/g_DW_ [41]. Research focusing on the phenolic composition of hawthorn leaves identified chlorogenic acid as one of the prevalent compounds, with contents in the leaves varying from 3 to 11 mg/g_DW_ [40].

Moreover, a comprehensive review of the chemical composition of hawthorn leaves reported that flavonoids are the main components of hawthorn leaves and that over 60 flavonoids and flavonoid glycosides have been identified, including vitexin derivatives [3].

Apigenin 8-*C*-glucoside (vitexin) derivatives, including *C*-glycosides and their rhamnosylated forms such as vitexin 2″-*O*-rhamnoside and isovitexin 2″-*O*-rhamnoside, are well-known compounds in *Crataegus* species. Vitexin and vitexin-2″-*O*-rhamnoside were found among the most abundant phenolic compounds in hawthorn leaves [33].

## 4. Conclusions

This study successfully optimized both solid–liquid extraction (SLE) and ultrasound-assisted extraction (UAE) methods for recovering phenolic compounds, flavonoids, and antioxidants from *Crataegus almaatensis* (hawthorn) leaves. Response surface methodology identified ethanol concentration as the most critical factor affecting extraction efficiency. UAE outperformed SLE by increasing total phenolic content by up to 16%, while requiring significantly less ethanol (40% vs. 75%), highlighting its effectiveness and sustainability. Temperature showed a significant non-linear effect, particularly in SLE, while ultrasound power and extraction time had minimal impact, supporting the potential for energy-efficient processing.

Phytochemical analysis (HPLC-DAD, LC-Q/TOF-MS) confirmed the presence of diverse bioactives, most notably chlorogenic acid and apigenin-8-C-glucoside-2′-rhamnoside, which contribute to the antioxidant and anti-inflammatory potential of hawthorn leaves. These findings establish UAE as a greener and more efficient technique for producing high-value extracts and support the future development of functional food, nutraceutical, and pharmaceutical applications. Overall, this research validates the utilization of the UAE process as a superior extraction method for the valorization of hawthorn leaves, offering both higher extraction efficiency and a greener approach through reduced solvent use compared to SLE. The results pave the way for the future development of standardized extracts and formulations from hawthorn leaves, contributing to a broader utilization of endemic plant species in food and health-promoting products. Further investigation into anti-inflammatory, cytoprotective effects on human cell lines will help guide the potential application of the optimized extracts.

## Figures and Tables

**Table 1:** Effect of the processing variables investigated on the response variables (TPC, TFC, FRAP, DPPH, and ABTS) in extracts from hawthorn leaves powder obtained after the SLE process.

Run	Variables				SLE				
	T (°C)	t (min)	EtOH (%)	S/L (g/mL)	TPC	TFC	FRAP	DPPH	ABTS
1	25	10	0	0.1	12.6 ± 0.1 _ab_	11.6 ± 0.3 _ab_	16.8 ± 0.0 _a_	54.3 ± 2.1 _de_	21.8 ± 0.5 _a_
2	25	90	0	0.1	13.2 ± 0.4 _abc_	12.3 ± 2.9 _ab_	22.7 ± 1.6 _b_	64.5 ± 1.3 _fgh_	27.5 ± 1.1 _b_
3	25	90	0	0.05	11.8 ± 0.2 _a_	12 ± 0.2 _ab_	24.2 ±1.6 _bc_	64.2 ± 3.1 _fgh_	30.5 ± 0.9 _bc_
4	25	10	80	0.1	31.9 ± 0.4 _ghi_	28.7 ± 0.1 _ghi_	58.9 ± 3.4 _ghi_	63.2 ± 3.7 _fgh_	77.6 ± 2.4 _op_
5	25	10	80	0.05	34.2 ± 0.7 _hil_	30.7 ± 1 _hil_	70.3 ± 1.3 _lm_	49.4 ± 0.4 _cd_	89.2 ± 2.7 _q_
6	25	50	40	0.075	32.5 ± 0.5 _hi_	25.6 ± 1.4 _gh_	64.9 ± 1.4 _lmn_	60.7 ± 2.4 _efgh_	80.4 ± 1.8 _p_
7	25	90	80	0.1	35.4 ± 0.4 _mn_	35.4 ± 3 _lmn_	56.9 ± 1.9 _gh_	66.6 ± 3 _h_	69.8 ± 1.5 _mn_
8	25	90	80	0.05	41.8 ± 0.5 _o_	38 ± 1.5 _def_	71.0 ± 1 _n_	53.4 ± 2.2 _de_	92.3 ± 1.3 _q_
9	25	10	0	0.05	18.9 ± 0.8 _e_	20 ± 0.9 _no_	28.7 ± 0.2 _cd_	54.5 ± 2.9 _de_	38.1 ± 0.8 _de_
10	47.5	50	40	0.1	24.2 ± 0.9 _f_	24.5 ± 1 _fg_	48.5 ± 1 _f_	66.5 ± 2.5 _h_	53.1 ± 1.3 _gh_
11	47.5	10	40	0.075	31.2 ± 0.3 _gh_	29.8 ± 1.3 _hi_	62.4 ± 1.8 _il_	64.9 ± 1.6 _gh_	77.1 ± 0.7 _op_
12	47.5	50	80	0.075	34 ± 0.2 _il_	30.7 ± 1 _hil_	56.0 ± 0.8 _gh_	67.6 ± 0.8 _h_	72.8 ± 1.0 _no_
13	47.5	50	40	0.05	31.7 ± 0.7 _ghi_	28.3 1.8 _ghi_	63.7 ± 2.4 _il_	67.1 ± 1.1 _h_	80.8 ± 2.1 _p_
14	47.5	50	0	0.075	12.1 ± 0.5 _a_	19.3 ± 0.4 _cde_	25.4 ± 1.3 _bcd_	58.2 ± 1.7 _efg_	33.0 ± 1.7 _c_
15	47.5	50	40	0.075	26 ± 1.3 _f_	28.3 ± 1 _ghi_	49.9 ± 0.5 _f_	65.9 ± 2.4 _h_	68.9 ± 0.9 _mn_
16	47.5	50	40	0.075	26.1 ± 1.5 _f_	26.5 ± 2 _gh_	49.2 ± 1.2 _f_	65.5 ± 2.9 _gh_	65.6 ± 1.5 _lm_
17	47.5	90	40	0.075	24.2 ± 0.4 _f_	23.5 ± 3 _efg_	46.7 ± 2.7 _f_	66.2 ± 3.8 _h_	55.7 ± 1.5 _hi_
18	70	90	80	0.1	29.2 ± 0.8 _g_	32.5 ± 1.2 _ilm_	48.9 ± 1 _f_	64.4 ± 2.1 _fgh_	60.6 ± 1.3 _il_
19	70	10	80	0.05	39.6 ± 0.2 _no_	42.3 ± 1.3 _o_	91.9 ± 3.4 _p_	63.3 ± 2.4 _fgh_	109.5 ± 2.4 _s_
20	70	10	80	0.1	33.8 ± 0.2 _hil_	37.9 ± 2.4 _no_	66.7 ± 2 _lmn_	63 ± 1.9 _fgh_	88.1 ± 2.6 _q_
21	70	50	40	0.075	41.7 ± 0.9 _o_	37 ± 1.4 _no_	85.2 ± 2.6 _o_	65.6 ± 1.8 _gh_	112.8 ± 2.4 _s_
22	70	90	80	0.05	36.9 ± 0.7 _mn_	38.9 ± 0.2 _no_	80.2 ± 0.4 _o_	64.1 ± 0.1 _fgh_	98.7 ± 1.5 _r_
23	70	10	0	0.05	15.9 ± 1 _cd_	14.5 ± 2.8 _bc_	30.1 ± 3.2 _d_	57.1 ± 3.9 _ef_	35.1 ± 1.2 _cd_
24	70	10	0	0.1	14.9 ± 1.1 _bc_	14.4 ± 0.5 _bc_	35.9 ± 2.5 _e_	44.2 ± 2.2 _bc_	45.7 ± 1.3 _f_
25	70	90	0	0.05	17.7 ± 0.2 _de_	15.4 ± 0.5 _bcd_	37.9 ± 0.3 _e_	40.2 ± 1.9 _ab_	50.6 ± 0.8 _fg_
26	70	90	0	0.1	11.8 ± 2 _a_	8.9 ± 2.7 _a_	27.4 ± 1.7 _bcd_	33.5 ± 1.9 _a_	40.6 ± 1.2 _e_

TPC (mg GAE/g_DM_), TFC (mg QE/g_DM_), FRAP (mg AAE/g_DM_), DPPH (%), ABTS (mg AAE/g_DM_). The results are expressed as mean ± standard deviation (*n* = 2 for factorial and axial points, *n* = 5 for central point). T (extraction temperature, °C), t (extraction time, min), EtOH (ethanol concentration in ethanol–water mixture, %, *v*/*v*); S/L (solid–liquid ratio, g/mL). Values with different lowercase letters within the same column are significantly different (*p* ≤ 0.05).

**Table 2:** Effect of the processing variables investigated on the response variables (TPC, TFC, FRAP, DPPH, and ABTS) in extracts from hawthorn leaves powder obtained after the UAE process.

Run	Variables				UAE				
	T (°C)	t (min)	EtOH (%)	P (W)	TPC	TFC	FRAP	DPPH	ABTS
1	40	60	40	100	39.6 ± 1.4 _bcdef_	39.7 ± 2.1 _bcdef_	75.1 ± 1.1 _fg_	59.8 ± 0.2 _efg_	96.7 ± 2.4 _gh_
2	40	30	70	400	35.6 ± 0.4 _ab_	35.9 ± 2.5 _ab_	68.2 ± 2.4 _cde_	59.1 ± 1.4 _ef_	80.7 ± 2.1 _ab_
3	40	60	70	400	36.5 ± 2.4 _abc_	36 ± 1 _ab_	63.3 ± 0.0 _a_	54.4 ± 0.4 _ab_	80.3 ± 1.0 _ab_
4	40	30	40	400	40.6 ± 0.1 _cdefhi_	38.5 ± 1.7 _abcde_	71.6 ± 1.7 _def_	58.6 ± 0.5 _cde_	89.1 ± 2.2 _def_
5	40	45	55	250	38.6 ± 0.8 _abcde_	38.3 ± 1.2 _abcde_	66.4 ± 0.4 _abc_	61.6 ± 0.5 _fghi_	80.3 ± 0.5 _ab_
6	40	60	70	100	34.9 ± 1.8 _a_	34.4 ± 0.0 _a_	64.8 ± 2 _abc_	60.1 ± 1 _fgh_	87.2 ± 1.6 _cde_
7	40	30	70	100	35.7 ± 0.7 _a_	37.1 ± 2.2 _abcd_	68.5 ± 0.9 _cde_	59 ± 0.9 _def_	89.0 ± 0.9 _def_
8	40	60	40	400	36.7 ± 1.9 _abcd_	35.6 ± 2.6 _abc_	63.3 ± 1 _a_	53.9 ± 0.8 _ab_	78.3 ± 1.3 _a_
9	40	30	40	100	37.8 ± 0.5 _abcd_	37.4 ± 1.8 _abcd_	64 ± 0.6 _ab_	62.9 ± 1.2 _hil_	83.2 ± 1.8 _bc_
10	55	45	55	250	40.2 ± 1.5 _cdefg_	38.9 ± 2.8 _abcde_	71.9 ± 1.4 _ef_	56.9 ± 0.6 _bcde_	90.5 ± 1.8 _ef_
11	55	30	55	250	40.8 ± 2.3 _cdefgh_	39.8 ± 1 _bcdef_	72.5 ± 1.6 _fg_	55.4 ± 0.9 _b_	92.3 ± 1.1 _fg_
12	55	45	55	100	44.1 ± 0.3 _ghi_	43.5 ± 1.5 _fgh_	76 ± 0.0 _gh_	56 ± 0.6 _bcd_	98.8 ± 1.0 _hi_
13	55	45	70	250	37.8 ± 0.3 _abcd_	37 ± 1.8 _abcd_	72.9 ± 0.3 _fg_	55.7 ± 1.2 _bc_	84.8 ± 0.8 _bcd_
14	55	45	40	250	42 ± 2.4 _efghi_	42.1 ± 1.3 _defgh_	79.8 ± 1.1 _hi_	54.2 ± 1.3 _ab_	100.7 ± 1.4 _hil_
15	55	45	55	400	43.1 ± 1 _fghi_	42.8 ± 0.9 _efgh_	80.7 ± 0.7 _il_	59.2 ± 1 _efg_	104.9 ± 1.2 _lm_
16	55	60	55	250	41 ± 2.9 _defgh_	38.1 ± 0.6 _abcd_	67.7 ± 0.5 _bcd_	55.2 ± 0.8 _b_	88.0 ± 0.9 _def_
17	70	60	40	400	51 ± 2.1 _m_	47 ± 2.5 _h_	89.6 ± 1 _n_	62.2 ± 1.6 _ghil_	106.5 ± 1.3 _mn_
18	70	30	40	100	44.2 ± 1 _ghi_	45.6 ± 2 g_h_	94.5 ± 0.8 _o_	61.1 ± 1.7 _fghi_	112.9 ± 1.0 _op_
19	70	30	70	100	40.1 ± 0.5 _cdefg_	38.4 ± 2 _abcde_	80.9 ± 1.3 _il_	51.5 ± 0.5 _a_	115.2 ± 0.7 _opq_
20	70	30	40	400	45.7 ± 2 _il_	40.6 ± 1.5 _bcdefg_	88 ± 1.2 _mn_	60.1 ± 1 _fgh_	104.4 ± 2.6 _lm_
21	70	60	70	100	41 ± 0.2 _fgh_	34 ± 0.5 _a_	84.5 ± 1 _lm_	59.8 ± 0.9 _efg_	110.8 ± 0.8 _no_
22	70	45	55	250	44.9 ± 1.4 _hil_	41.5 ± 0.4 _cdefg_	88.9 ± 0.9 _n_	61.3 ± 0.9 _fghi_	115.6 ± 1.2 _pq_
23	70	60	70	400	40.5 ± 0.9 _cdefg_	38.9 ± 0.3 _abcde_	87 ± 0.7 _mn_	63.6 ± 0.5 _il_	103.1 ± 1.1 _ilm_
24	70	60	40	100	48.9 ± 0.9 _lm_	45.5 ± 1.7 _gh_	95.3 ± 2.8 _o_	65.2 ± 1 _l_	117.9 ± 1.6 _q_
25	70	30	70	400	39.3 ± 1.5 _abcdef_	37.1 ± 0.2 _abcd_	80.1 ± 1.5 _i_	59.2 ± 0.7 _efg_	104.1 ± 2.0 _lm_

TPC (mg GAE/g_DM_), TFC (mg QE/g_DM_), FRAP (mg AAE/g_DM_), DPPH (%), ABTS (mg AAE/g_DM_). The results are expressed as mean ± standard deviation (*n* = 2 for factorial and axial points, *n* = 5 for central point). T (extraction temperature, °C), t (extraction time, min), EtOH (ethanol concentration in ethanol–water mixture, %, *v*/*v*); P (US power, W). Values with different lowercase letters within the same column are significantly different (*p* ≤ 0.05).

**Table 3:** Coefficients and ANOVA parameters of the reduced second-order polynomial model for TPC, TFC, and antioxidant activity (FRAP, DPPH, and ABTS) in extracts from hawthorn leaves powder obtained after the SLE process.

SLE							
TPC (mgGAE/g_DM_)							
	Coefficients	Sum of Squares	df	Mean Square	F-Value	*p*-Value	Contribution (%)
α_0_	38.73962	-	-	-	-	*	-
α_1_ (T)	−1.07297	38.03	1	38.03	5.41	*	1.74
α_3_ (EtOH)	0.74447	1736.46	1	1736.46	247.04	***	79.52
α_4_ (S/L ratio)	−85.44843	82.14	1	82.14	11.69	**	3.76
α_11_ (T × T)	0.011974	120.45	1	120.45	17.14	***	5.52
α_33_ (EtOH × EtOH)	−0.006237	326.37	1	326.37	46.43	***	14.95
Model	-	2183.54	5	436.71	62.13	***	-
Lack of fit	-	140.58	19	7.40	1.45	ns	-
R^2^	0.940						
Adjusted R^2^	0.924						
Predicted R^2^	0.887						
Adequate precision	23.002						
TFC (mgQE/g_DM_)							
α_0_	5.69088	-	-	-	-	*	-
α_3_ (EtOH)	0.298550	2567.00	1	2567.00	85.88	***	94.14
α_12_ (T × t)	−0.003347	145.19	1	145.19	4.86	*	5.32
Model	-	2726.71	4	681.68	22.81	**	-
Lack of fit	-	626.02	20	31.30	1.48	ns	-
R^2^	0.883						
Adjusted R^2^	0.878						
Predicted R^2^	0.788						
Adequate precision	19.169						
FRAP (mgAAE/g_DM_)							
α_0_	73.91293	-	-	-	-	**	-
α_1_ (T)	−2.38291	446.29	1	446.29	9.45	**	4.6
α_3_ (EtOH)	1.81099	6884.20	1	6884.20	4.86	***	70.98
α_4_ (S/L ratio)	−96.66875	738.95	1	738.95	15.64	**	7.62
α_34_ (EtOH × S/L ratio)	−3.99053	254.79	1	254.79	5.39	*	2.63
α_11_ (T × T)	0.02741	631.27	1	631.27	13.36	**	6.50
α_33_ (EtOH × EtOH)	−0.012785	1371.54	1	1371.54	29.03	***	14.14
Model	-	9699.39	6	1616.57	34.21	***	-
Lack of fit	-	897.40	18	49.86	0.42	ns	-
R^2^	0.915						
Adjusted R^2^	0.889						
Predicted R^2^	0.835						
Adequate precision	20.890						
DPPH (%)							
α_0_	50.31451	-	-	-	-	***	-
α_3_ (EtOH)	−0.38078	392.62	1	392.62	8.34	***	36.19
α_12_ (T × t)	−0.00366	173.65	1	173.65	7.69	*	16
α_13_ (T × EtOH)	0.005884	448.65	1	448.65	12.53	***	41.36
Model	-	1084.82	5	216.96	4.61	***	-
Lack of fit	-	941.73	19	49.56	2.80	ns	-
R^2^	0.870						
Adjusted R^2^	0.820						
Predicted R^2^	0.795						
Adequate precision	13.187						
ABTS (mgAAE/g_DM_)							
α_0_	109.9680	-	-	-	-	**	-
α_1_ (T)	−3.093153	728.35	1	728.35	6.83	*	5
α_3_ (EtOH)	1.890647	10,546.36	1	105,46.36	98.92	***	72.51
α_4_ (S/L ratio)	−311.11111	1088.89	1	1088.89	10.21	**	7.49
α_11_ (T × T)	0.035535	1060.80	1	1060.80	9.95	**	7.29
α_33_ (EtOH × EtOH)	−0.016069	2166.65	1	2166.65	20.32	**	14.90
Model	-	14,543.80	5	2908.76	27.28	***	-
Lack of fit	-	2126.88	19	111.94	1.56	ns	-
R^2^	0.942						
Adjusted R^2^	0.910						
Predicted R^2^	0.838						
Adequate precision	16.237						

ns not significant for *p* > 0.05. * Significant for *p* ≤ 0.05; ** significant for *p* ≤ 0.01; *** significant for *p* ≤ 0.001.

**Table 4:** Coefficients and ANOVA parameters of the reduced second-order polynomial model for TPC, TFC, and antioxidant activity (FRAP, DPPH, and ABTS) in extracts from hawthorn leaves powder obtained after the UAE process.

UAE							
TPC (mgGAE/g_DM_)							
	Coefficients	Sum of Squares	df	Mean Square	F-Value	*p*-Value	Contribution %
α_0_	3.81775	-	-	-	-	**	-
α_1_ (T)	0.297404	191.07	1	191.07	195.33	***	54.75
α_3_ (EtOH)	1.25735	93.51	1	93.51	95.59	***	28.26
α_12_ (T × t)	0.003581	10.38	1	10.38	10.62	**	3.14
α_13_ (T × EtOH)	−0.004388	15.59	1	15.59	15.94	**	4.71
α_33_ (EtOH × EtOH)	−0.010618	18.30	1	18.30	18.71	**	5.56
α_44_ (Power × Power)	0.000060	5.92	1	5.92	6.05	*	1.79
Model	-	330.87	8	41.36	42.48	***	-
Lack of fit	-	94.5	10	9.45	1.05	ns	-
R^2^	0.955						
Adjusted R^2^	0.932						
Predicted R^2^	0.881						
Adequate precision	21.866						
TFC (mgQE/g_DM_)							
α_0_	22.50699	-	-	-	-	**	-
α_1_ (T)	0.476451	71.87	1	71.87	11.09	**	30.62
α_3_ (EtOH)	0.164074	130.01	1	130.01	20.07	***	55.39
α_13_ (T × EtOH)	−0.006241	31.55	1	31.55	4.87	*	13.44
Model	-	234.73	4	58.68	12.48	***	-
Lack of fit	-	16.42	6	2.74	1.87	ns	-
R^2^	0.910						
Adjusted R^2^	0.875						
Predicted R^2^	0.793						
Adequate precision	13.445						
FRAP (mgAAE/g_DM_)							
α_0_	48.79112	-	-	-	-	***	-
α_1_ (T)	0.68029	1874.34	1	1874.34	97.75	***	92.79
α_3_ (EtOH)	−0.189417	145.31	1	145.31	7.58	**	7.19
Model	-	2019.94	4	504.99	26.33	***	-
Lack of fit	-	74.5	10	7.45	1.250	ns	-
R^2^	0.940						
Adjusted R^2^	0.909						
Predicted R^2^	0.857						
Adequate precision	17.279						
DPPH (%)							
α_0_	136.43547	-	-	-	-	**	-
α_12_ (T × t)	0.00842	57.43	1	57.43	12.91	***	27.38
α_14_ (T × Power)	0.000657	34.50	1	34.50	7.76	**	14.45
α_34_ (EtOH × Power)	0.000559	25.33	1	25.33	5.69	*	12.08
α_11_ (T × T)	0.015732	63.15	1	63.15	14.20	***	30.10
Model	-	209.77	8	26.22	6.12	**	-
Lack of fit	-	20.23	6	3.37	0.101	ns	-
R^2^	0.886						
Adjusted R^2^	0.857						
Predicted R^2^	0.778						
Adequate precision	18.105						
ABTS (mgAAE/g_DM_)							
α_0_	56.21941	-	-	-	-	***	-
α_1_ (T)	0.83593	2830.03	1	2830.03	87.23	***	93.34
α_4_(Power)	−0.02233	202.00	1	202.00	6.23	*	6.67
Model	-	3032.03	2	1516.02	46.73	***	-
Lack of fit	-	187.18	6	31.20	0.065	ns	-
R^2^	0.890						
Adjusted R^2^	0.822						
Predicted R^2^	0.797						
Adequate precision	16.105						

ns not significant for *p* > 0.05. * Significant for *p* ≤ 0.05; ** significant for *p* ≤ 0.01; *** significant for *p* ≤ 0.001.

**Table 5:** TPC, TFC, FRAP, DPPH, and ABTS predicted and experimental values of hawthorn leaves powder extracts obtained after SLE and UAE processes at the optimized conditions.

	SLE		UAE		% Deviation	
Optimal processing conditions	T: 70 °C t: 34 min EtOH%: 75% S/L ratio: 0.05 g/mL		T: 70 °C t: 44 min EtOH%: 40% S/L ratio: 0.05 g/mL US power: 100 W			
	Predicted value	Experimental value	Predicted value	Experimental value	SLE	UAE
TPC (mgGAE/g_DM_)	39.75 ± 0.30 _a_	41.28 ± 0.90 _a_	47.75 ± 0.85 _b_	48.00 ± 0.85 _b_	3.85	0.52
TFC (mgQE/g_DM_)	39.74 ± 0.10 _a_	40.64 ± 0.63 _a_	46.82 ± 0.85 _b_	43.68 ± 0.77 _b_	2.26	6.71
FRAP (mgAAE/g_DM_)	86.97 ± 1.20 _a_	91.98 ± 1.48 _a_	89.77 ± 0.85 _b_	98.61 ± 1.15 _b_	5.76	9.84
DPPH (%)	67.83 ± 0.95 _b_	64.26 ± 1.25 _a_	64.49 ± 0.85 _a_	66.73 ± 1.10 _a_	5.27	3.47
ABTS (mgAAE/g_DM_)	106.08 ± 1.30 _a_	109.08 ± 1.70 _a_	112.50 ± 0.85 _b_	118.50 ± 1.35 _b_	2.83	5.33

Values with different lowercase letters within the same line for predicted values and experimental values are significantly different (*p* ≤ 0.05).

**Table 6:** Concentrations (mg/g_DM_) of catechin, chlorogenic acid, epicatechin, and apigenin-8-C-glucoside-2′-rhamnoside identified in hawthorn leaf powder extracts obtained after SLE and UAE processes at the optimized conditions.

N.	Compound	Concentration	
		SLE	UAE
3	Catechin	0.04 ± 0 _a_	0.04 ± 0 _b_
4	Chlorogenic acid	1.68 ± 0.2 _a_	2.42 ± 0.3 _b_
6	Epicatechin	0.06 ± 0 _a_	0.08 ± 0 _b_
11	Apigenin-8-*C*-glucoside-2′-rhamnoside	1.28 ± 0.1 _a_	1.75 ± 0.2 _b_

Values with different lowercase letters within the same line are significantly different (*p* ≤ 0.05).

**Table 7:** High-resolution mass spectrometry (LC-Q/TOF)—based assignment of chromatographic peaks in the extract obtained after SLE under optimized conditions (temperature of 70 °C, solvent 75% ethanol–water mixture, time of 34 min, and S/L ratio of 0.05 g/mL).

N.	Compound	Formula	[M-H]- Theoretical (*m*/*z*)	[M-H]- Measured (*m*/*z*)	Error (ppm)
1	Neochlorogenic acid	C_16_H_18_O_9_	353.0872	353.0867	−1.42
2	1-*O*-galloyl-l-rhamnose	C_13_H_16_O_9_	315.0716	315.0714	−0.63
3	Catechin	C_15_H_14_O_6_	289.0712	289.0714	0.69
4	Chlorogenic acid	C_16_H_18_O_9_	353.0872	353.0869	−0.85
5	Cryptochlorogenic acid	C_16_H_18_O_9_	353.0872	353.0881	2.55
6	Epicatechin	C_15_H_14_O_6_	289.0712	289.0719	2.42
7	Procyanidin C1	C_45_H_38_O_18_	865.1979	865.1990	1.27
8	Apigenin-6-*C*-glucoside-8-*C*-arabinoside	C_26_H_28_O_14_	563.1400	563.1411	1.95
9	Luteolin-7-*O*-glucoside	C_21_H_20_O_11_	447.0927	447.0932	1.12
10	Isovitexin-2″-*O*-arabinoside	C_26_H_28_O_14_	563.1400	563.1398	−0.36
11	Apigenin-8-*C*-glucoside-2′-rhamnoside	C_27_H_30_O_14_	577.1557	577.1565	1.39
12	Isovitexin (apigenin-6-*C*-glucoside)	C_21_H_20_O_10_	431.0978	431.0975	−0.70
13	Vitexin (apigenin-8-*C*-glucoside)	C_21_H_20_O_10_	431.0978	431.0982	0.93
14	Hyperoside (quercetin-3-*O*-galactoside)	C_21_H_20_O_12_	463.0876	463.0871	−1.08
15	Isoquercitrin (quercetin-3-*O*-glucoside)	C_21_H_19_O_12_	463.0876	463.0877	0.22
16	Nothofagin	C_21_H_24_O_10_	435.1291	435.1297	1.38
17	Quercetin 3-*O*-(6″-*O*-malonyl)-glucoside	C_24_H_22_O_15_	549.0880	549.0888	1.46
18	Cinchonain 1A	C_24_H_20_O_9_	451.1029	451.1036	1.55

## Data Availability

The data presented in this study are available within the article. The original data that support the findings of this study are available from the corresponding author upon reasonable request.

## Supplementary Materials

The following supporting information can be downloaded at: https://www.mdpi.com/article/10.3390/antiox14081003/s1. Table S1. Reduced second-order polynomial model equations for TPC, TFC, and antioxidant activity (FRAP, DPPH, and ABTS) in extracts from hawthorn leaves powder obtained after SLE process. Table S2. Reduced second-order polynomial model equations for TPC, TFC, and antioxidant activity (FRAP, DPPH, and ABTS) in extracts from hawthorn leaves powder obtained after UAE process. Table S3. Coefficients of the full second-order polynomial model for TPC, TFC, and antioxidant activity (FRAP and DPPH) in extracts from hawthorn leaves powder obtained after SLE process. Table S4. Coefficients of the full second-order polynomial model for TPC, TFC, and antioxidant activity (FRAP and DPPH) in extracts from hawthorn leaves powder obtained after UAE process. Figure S1. Correlation of predicted vs. actual values of TPC, TFC, FRAP, DPPH, and ABTS of extracts obtained after SLE process of hawthorn leaves powder. Figure S2. Correlation of predicted vs. actual values of TPC, TFC, FRAP, DPPH, and ABTS of extracts obtained after UAE process of hawthorn leaves powder. Figure S3. Normal plots of residuals for TPC, TFC, FRAP, DPPH, and ABTS of extracts obtained after SLE process of hawthorn leaves powder. Figure S4. Normal plots of residuals for TPC, TFC, FRAP, DPPH, and ABTS of extracts obtained after UAE process of hawthorn leaves powder.
