# Supplementary material for: Optimization of Conventional and Ultrasound-Assisted Extraction to Maximize Recovery of Total Phenolic Content and In Vitro Antioxidant Activity from Crataegus almaatensis Leaves
Source: Antioxidants (Basel). 2025 Aug 16;14(8):1003. doi: 10.3390/antiox14081003 (PMC12382680; doi:10.3390/antiox14081003)

Supplementary Material

Table S1. Reduced second-order polynomial model equations for TPC, TFC, and antioxidant activity (FRAP, DPPH, and ABTS) in extracts from hawthorn leaves powder obtained after SLE process.

SLE			
**Response variable (Y)**	**Equation**	***p* value**	**R^2**
TPC (mgGAE/g_{DM})	$TPC = 38.73962 - 1.07297 * T + 0.74447 * EtOH - 85.44843 * S/L + 0.011974 * T^2 - 0.006237 * EtOH^2$	<0.0001	0.940
TFC (mgQE/g_{DM})	$TFC = 5.69088 + 0.298550 * EtOH - 0.003347 * Txt$	<0.01	0.883
FRAP (mgAAE/g_{DM})	$FRAP = 73.91293 - 2.38291 * T + 1.81099 * EtOH - 96.66875 * S/L - 3.99053 * EtOHxS/L + 0.02741 * T^2 - 0.012785 * EtOH^2$	<0.0001	0.915
DPPH (%)	$DPPH = 50.31451 - 0.38078 * EtOH - 0.00366 * Txt +* 0.005884\ TxEtOH$	<0.0005	0.870
ABTS (mgAAE/g_{DM})	$ABTS = 109.9680 - 3.093153 * T + 1.890647 * EtOH - 311.11111 * S/L + 0.035535 * T^2 - 0.016069 * EtOH^2$	<0.0001	0.942

Table S2. Reduced second-order polynomial model equations for TPC, TFC, and antioxidant activity (FRAP, DPPH, and ABTS) in extracts from hawthorn leaves powder obtained after UAE process.

UAE			
**Response variable (Y)**	**Equation**	***p* value**	**R^2**
TPC (mgGAE/g_{DM})	$TPC = 3.81775 + 0.297404 * T + 1.25735 * EtOH + 0.003581 * Txt - 0.004388 * TxEtOH - 0.010618 * EtOH^2 + 0.000060 * P^2$	<0.0001	0.955
TFC (mgQE/g_{DM})	$TFC = 23.31672 + 0.47645 * T + 0.16407 * EtOH - 0.006241 * TxEtOH$	<0.0001	0.910
FRAP (mgAAE/g_{DM})	$FRAP = 48.79112 + 0.68029 * T - 0.189417 * EtOH$	<0.0001	0.940
DPPH (%)	$DPPH = 136.43547 + 0.00842 * Txt + 0.000657 * TxP + 0.000559\ EtOHxP + 0.015732 * T^2$	<0.001	0.886
ABTS (mgAAE/g_{DM})	$ABTS = 56.21941 + 0.83593 * T - 0.02233 * P$	<0.0001	0.890

Table S3. Coefficients of the full second-order polynomial model for TPC, TFC, and antioxidant activity (FRAP and DPPH) in extracts from hawthorn leaves powder obtained after SLE process.

**Coefficients**	**SLE**							
	TPC ($mgGAE/g_{DW}$)		TFC ($mgQE/g_{DW}$)		FRAP ($mgAAE/g_{DW}$)		DPPH (%)	
α_0	+22.52770		+4.24624		+42.24410		+31.52569	
α_1 (T)	-1.23630	*	-0.189755	ns	-2.54636	**	+1.01299	*
α_2 (time)	+0.131415	ns	+0.098407	ns	+0.408800	ns	+0.226497	ns
α_3 (EtOH)	+0.728243	***	+0.253981	***	+1.77300	***	-0.195578	***
α_4 (S/L ratio)	+404.26905	**	+439.93412	ns	+668.08273	**	+228.50166	ns
α_{12} (T x t)	-0.000274	ns	-0.003347	*	-0.002106	ns	-0.003660	**
α_{13} (T x EtOH)	-0.000054	ns	+0.001211	ns	-0.000586	ns	+0.005884	***
α_{14} (T x S/L)	-1.10953	ns	-3.18686	ns	-2.50385	ns	-5.19740	**
α_{23} (t x EtOH)	-0.000144	ns	+0.000981	ns	-0.001241	ns	+0.000681	ns
α_{24} (t x S/L)	-0.268237	ns	+1.47861	ns	-0.916293	ns	+0.412088	ns
α_{34} (EtOH x S/L)	-0.585167	ns	-0.037689	ns	-3.99053	*	+2.90751	**
α_{11} (T x T)	+0.014737	***	+0.006165	ns	+0.032465	**	-0.008029	ns
α_{22} (t x t)	-0.001200	ns	-0.000965	ns	-0.002538	ns	-0.001064	ns
α_{33} (EtOH x EtOH)	-0.005363	***	-0.000740	ns	-0.011186	***	-0.002741	ns
α_{44} (S/L x S/L)	-2667.9752	ns	-2847.00210	ns	-4000.0275	ns	-686.55154	ns
p value of the model	<0.0001	***	<0.01		<0.0001	***	<0.0005	***
R^2	0.965		0.883		0.945		0.915	

ns not significant for p > 0.05

*Significant for p ≤ 0.05; **significant for p ≤ 0.01; ***significant for p ≤ 0.001

Table S4. Coefficients of the full second-order polynomial model for TPC, TFC, and antioxidant activity (FRAP and DPPH) in extracts from hawthorn leaves powder obtained after UAE process.

**Coefficients**	**UAE**							
	TPC (mgGAE/g_{DW})		TFC (mgQE/g_{DW})		FRAP (mgAAE/g_{DW})		DPPH (%)	
α_0	-0.893321		-11.25449		+61.04745		+119.06459	
α_1 (T)	+0.239161	***	+0.875893	**	-0.515570	***	-2.65979	*
α_2 (time)	+0.128986	ns	+0.910345	ns	+1.78693	ns	+0.051685	ns
α_3 (EtOH)	+1.18965	***	+0.771526	***	-0.579035	**	+0.671729	*
α_4 (Power)	-0.018217	ns	-0.102177	ns	-0.078926	ns	-0.073275	ns
α_{12} (T x t)	+0.003581	**	+0.001917	ns	+0.005218	ns	+0.008420	***
α_{13} (T x EtOH)	-0.004388	**	-0.006241	*	-0.007206	ns	-0.003308	ns
α_{14} (T x Power)	-0.000114	ns	+0.000242	ns	-0.000132	ns	+0.000653	**
α_{23} (t x EtOH)	-0.000152	ns	-0.004713	ns	-0.000879	ns	+0.002971	ns
α_{24} (t x Power)	-0.000117	ns	+0.000280	ns	-0.000462	ns	-0.000370	ns
α_{34} (EtOH x Power)	-0.000065	ns	+0.000286	ns	+0.000455	ns	+0.000559	**
α_{11} (T x T)	+0.000789	ns	-0.004966	ns	+0.012641	ns	+0.021398	***
α_{22} (t x t)	-0.002983	ns	-0.008984	ns	-0.021132	*	-0.006211	ns
α_{33} (EtOH x EtOH)	-0.009792	**	-0.004243	ns	+0.006471	ns	-0.007456	ns
α_{44} (Power x Power)	+0.000069	*	+0.000118	ns	+0.000155	ns	+0.000043	ns
p value of the model	<0.0001	***	<0.01		<0.0004	***	<0.01	***
R^2	0.965		0.844		0.935		0.915	

ns not significant for p > 0.05

*Significant for $p \leq 0.05$; **significant for $p \leq 0.01$; ***significant for $p \leq 0.001$

Figure S1. Correlation of predicted vs. actual values of TPC, TFC, FRAP, DPPH, and ABTS of extracts obtained after SLE process of hawthorn leaves powder.

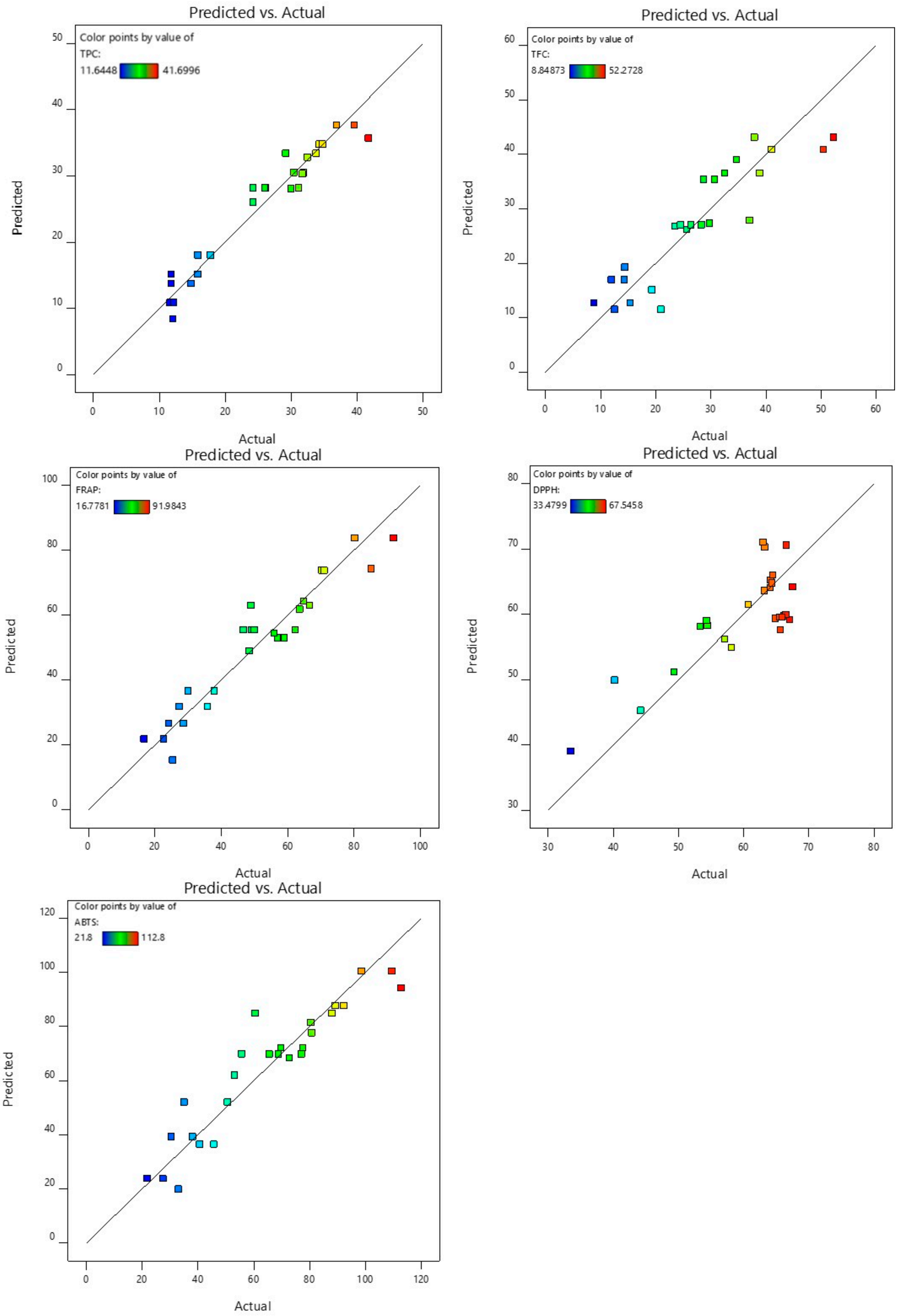

Figure S2. Correlation of predicted vs. actual values of TPC, TFC, FRAP, DPPH, and ABTS of extracts obtained after UAE process of hawthorn leaves powder.

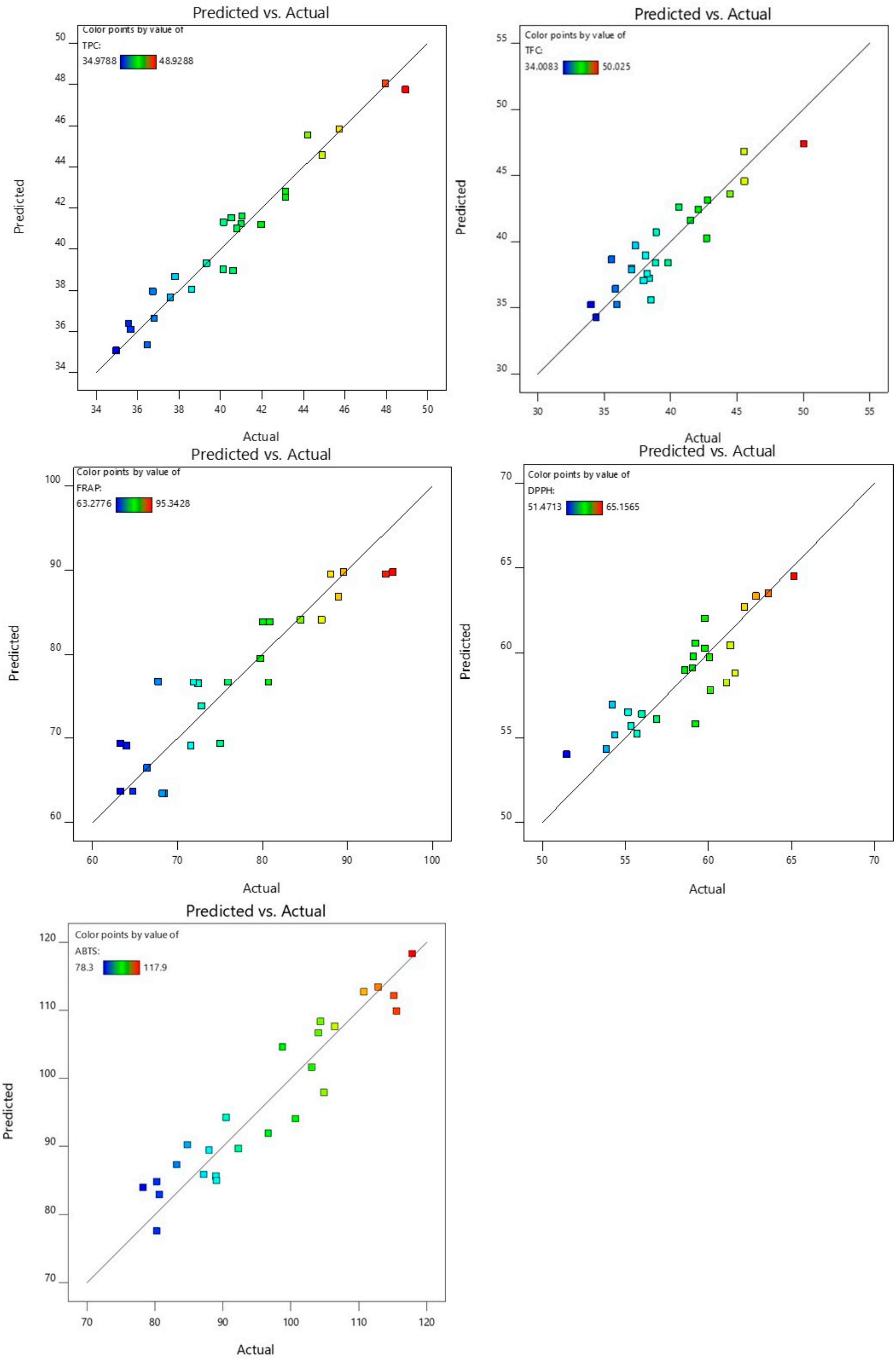

Figure S3. Normal plots of residuals for TPC, TFC, FRAP, DPPH, and ABTS of extracts obtained after SLE process of hawthorn leaves powder.

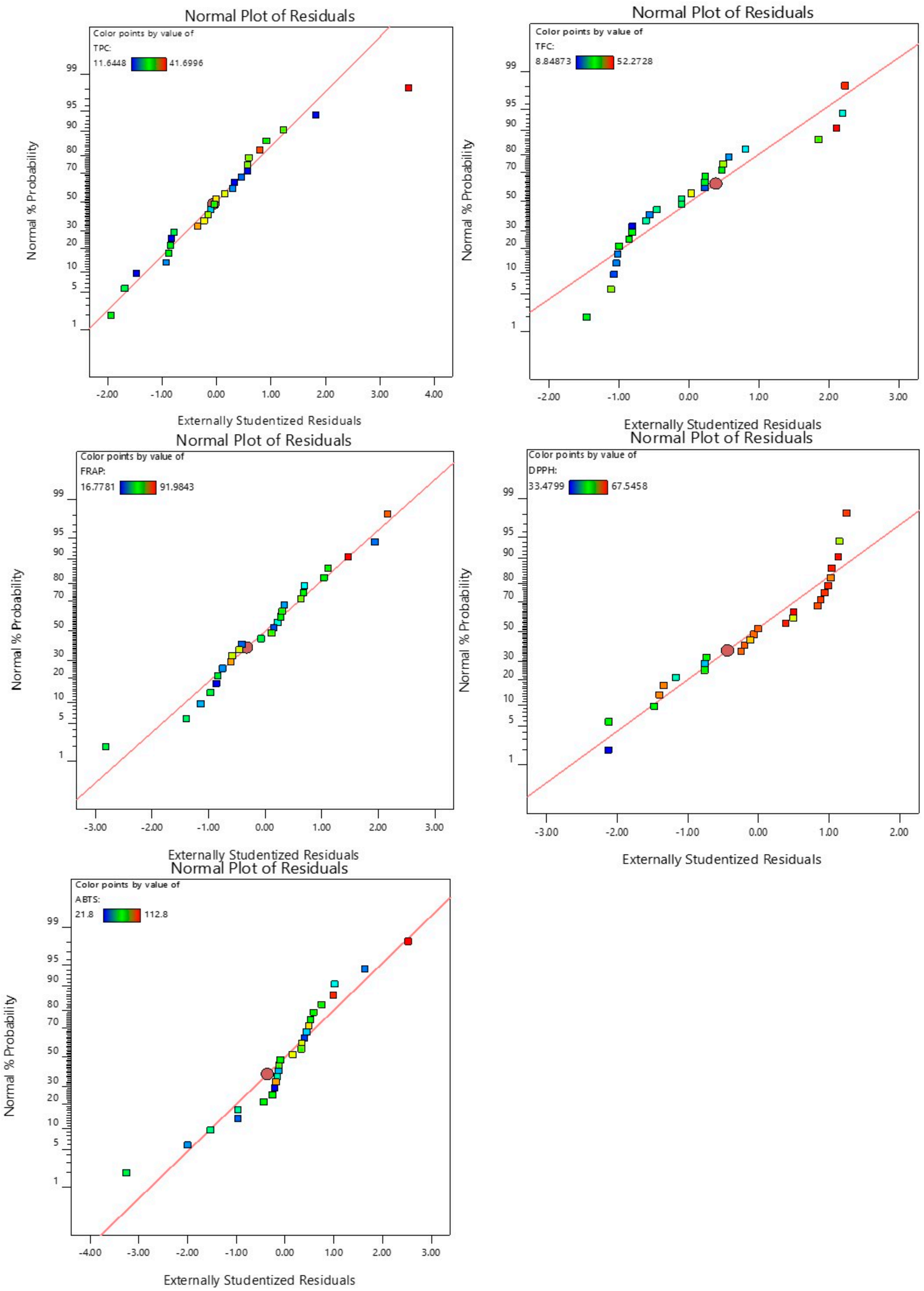

Figure S4. Normal plots of residuals for TPC, TFC, FRAP, DPPH, and ABTS of extracts obtained after UAE process of hawthorn leaves powder.

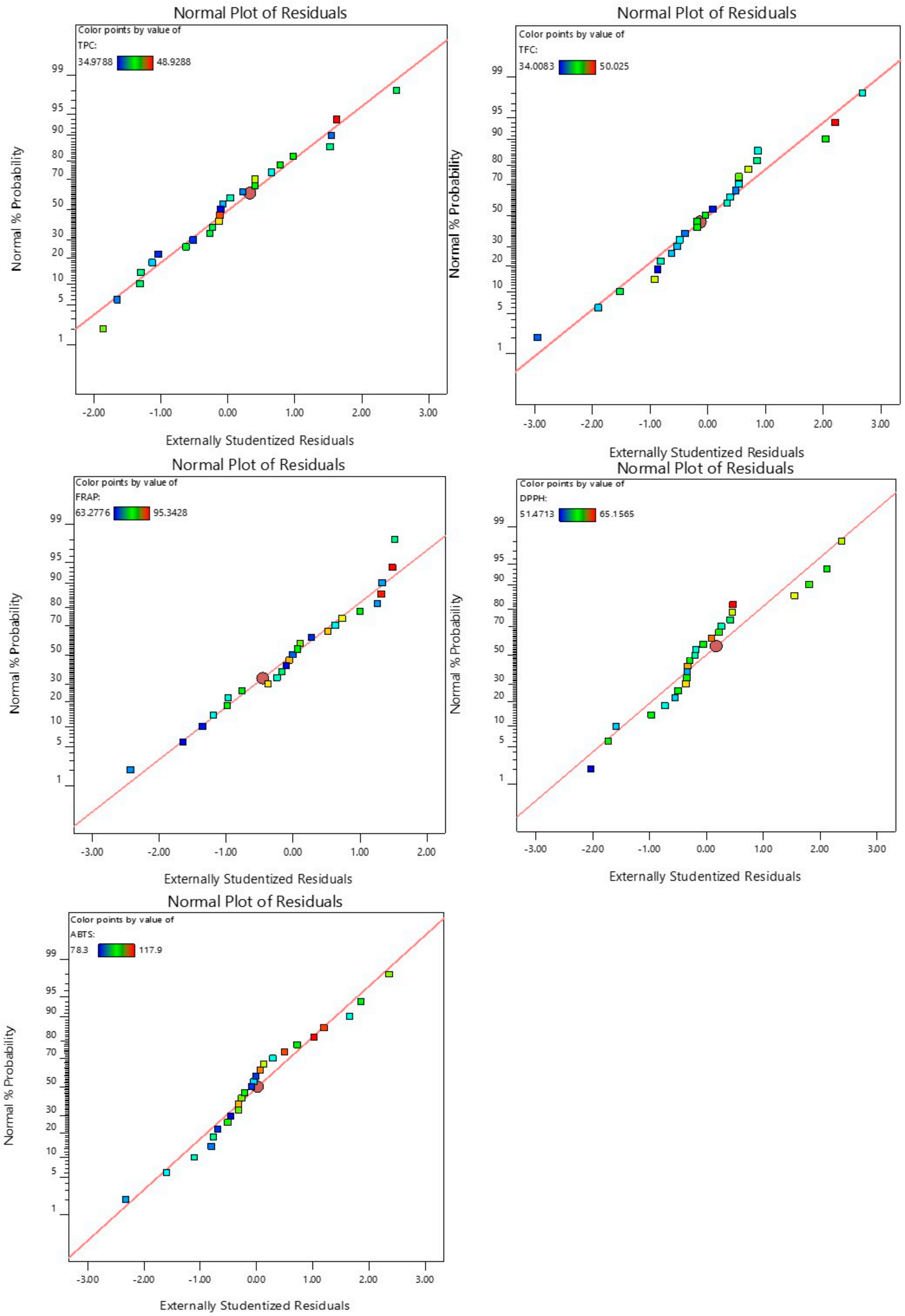

Supplement: Supplementary file 1 [file antioxidants-14-01003-s001.zip › antioxidants-3750368-supplementary.pdf]
